# Social and moral psychology of COVID-19 across 69 countries

**DOI:** 10.1038/s41597-023-02080-8

**Published:** 2023-05-11

**Authors:** Flavio Azevedo, Tomislav Pavlović, Gabriel G. Rêgo, F. Ceren Ay, Biljana Gjoneska, Tom W. Etienne, Robert M. Ross, Philipp Schönegger, Julián C. Riaño-Moreno, Aleksandra Cichocka, Valerio Capraro, Luca Cian, Chiara Longoni, Ho Fai Chan, Jay J. Van Bavel, Hallgeir Sjåstad, John B. Nezlek, Mark Alfano, Michele J. Gelfand, Michèle D. Birtel, Aleksandra Cislak, Patricia L. Lockwood, Koen Abts, Elena Agadullina, John Jamir Benzon Aruta, Sahba Nomvula Besharati, Alexander Bor, Becky L. Choma, Charles David Crabtree, William A. Cunningham, Koustav De, Waqas Ejaz, Christian T. Elbaek, Andrej Findor, Daniel Flichtentrei, Renata Franc, June Gruber, Estrella Gualda, Yusaku Horiuchi, Toan Luu Duc Huynh, Agustin Ibanez, Mostak Ahamed Imran, Jacob Israelashvili, Katarzyna Jasko, Jaroslaw Kantorowicz, Elena Kantorowicz-Reznichenko, André Krouwel, Michael Laakasuo, Claus Lamm, Caroline Leygue, Ming-Jen Lin, Mohammad Sabbir Mansoor, Antoine Marie, Lewend Mayiwar, Honorata Mazepus, Cillian McHugh, John Paul Minda, Panagiotis Mitkidis, Andreas Olsson, Tobias Otterbring, Dominic J. Packer, Anat Perry, Michael Bang Petersen, Arathy Puthillam, Tobias Rothmund, Hernando Santamaría-García, Petra C. Schmid, Drozdstoy Stoyanov, Shruti Tewari, Bojan Todosijević, Manos Tsakiris, Hans H. Tung, Radu G. Umbres, Edmunds Vanags, Madalina Vlasceanu, Andrew Vonasch, Meltem Yucel, Yucheng Zhang, Mohcine Abad, Eli Adler, Narin Akrawi, Hamza Alaoui Mdarhri, Hanane Amara, David M. Amodio, Benedict G. Antazo, Matthew Apps, Mouhamadou Hady Ba, Sergio Barbosa, Brock Bastian, Anton Berg, Maria P. Bernal-Zárate, Michael Bernstein, Michał Białek, Ennio Bilancini, Natalia Bogatyreva, Leonardo Boncinelli, Jonathan E. Booth, Sylvie Borau, Ondrej Buchel, C. Daryl Cameron, Chrissie F. Carvalho, Tatiana Celadin, Chiara Cerami, Hom Nath Chalise, Xiaojun Cheng, Kate Cockcroft, Jane Conway, Mateo Andres Córdoba-Delgado, Chiara Crespi, Marie Crouzevialle, Jo Cutler, Marzena Cypryańska, Justyna Dabrowska, Michael A. Daniels, Victoria H. Davis, Pamala N. Dayley, Sylvain Delouvée, Ognjan Denkovski, Guillaume Dezecache, Nathan A. Dhaliwal, Alelie B. Diato, Roberto Di Paolo, Marianna Drosinou, Uwe Dulleck, Jānis Ekmanis, Arhan S. Ertan, Hapsa Hossain Farhana, Fahima Farkhari, Harry Farmer, Ali Fenwick, Kristijan Fidanovski, Terry Flew, Shona Fraser, Raymond Boadi Frempong, Jonathan A. Fugelsang, Jessica Gale, E. Begoña Garcia-Navarro, Prasad Garladinne, Oussama Ghajjou, Theofilos Gkinopoulos, Kurt Gray, Siobhán M. Griffin, Bjarki Gronfeldt, Mert Gümren, Ranju Lama Gurung, Eran Halperin, Elizabeth Harris, Volo Herzon, Matej Hruška, Guanxiong Huang, Matthias F. C. Hudecek, Ozan Isler, Simon Jangard, Frederik J. Jorgensen, Frank Kachanoff, John Kahn, Apsara Katuwal Dangol, Oleksandra Keudel, Lina Koppel, Mika Koverola, Emily Kubin, Anton Kunnari, Yordan Kutiyski, Oscar Moreda Laguna, Josh Leota, Eva Lermer, Jonathan Levy, Neil Levy, Chunyun Li, Elizabeth U. Long, Marina Maglić, Darragh McCashin, Alexander L. Metcalf, Igor Mikloušić, Soulaimane El Mimouni, Asako Miura, Juliana Molina-Paredes, César Monroy-Fonseca, Elena Morales-Marente, David Moreau, Rafał Muda, Annalisa Myer, Kyle Nash, Tarik Nesh-Nash, Jonas P. Nitschke, Matthew S. Nurse, Yohsuke Ohtsubo, Victoria Oldemburgo de Mello, Cathal O’Madagain, Michal Onderco, M. Soledad Palacios-Galvez, Jussi Palomöki, Yafeng Pan, Zsófia Papp, Philip Pärnamets, Mariola Paruzel-Czachura, Zoran Pavlović, César Payán-Gómez, Silva Perander, Michael Mark Pitman, Rajib Prasad, Joanna Pyrkosz-Pacyna, Steve Rathje, Ali Raza, Kasey Rhee, Claire E. Robertson, Iván Rodríguez-Pascual, Teemu Saikkonen, Octavio Salvador-Ginez, Gaia C. Santi, Natalia Santiago-Tovar, David Savage, Julian A. Scheffer, David T. Schultner, Enid M. Schutte, Andy Scott, Madhavi Sharma, Pujan Sharma, Ahmed Skali, David Stadelmann, Clara Alexandra Stafford, Dragan Stanojević, Anna Stefaniak, Anni Sternisko, Augustin Stoica, Kristina K. Stoyanova, Brent Strickland, Jukka Sundvall, Jeffrey P. Thomas, Gustav Tinghög, Benno Torgler, Iris J. Traast, Raffaele Tucciarelli, Michael Tyrala, Nick D. Ungson, Mete S. Uysal, Paul A. M. Van Lange, Jan-Willem van Prooijen, Dirk van Rooy, Daniel Västfjäll, Peter Verkoeijen, Joana B. Vieira, Christian von Sikorski, Alexander Cameron Walker, Jennifer Watermeyer, Erik Wetter, Ashley Whillans, Katherine White, Rishad Habib, Robin Willardt, Michael J. A. Wohl, Adrian Dominik Wójcik, Kaidi Wu, Yuki Yamada, Onurcan Yilmaz, Kumar Yogeeswaran, Carolin-Theresa Ziemer, Rolf A. Zwaan, Paulo S. Boggio, Waldir M. Sampaio

**Affiliations:** 1 Department of Psychology, University of Cambridge, Cambridge, England; 2 Institute of Communication Science, Friedrich-Schiller University Jena, Jena, Germany; 3 Institute of Social Sciences Ivo Pilar, Zagreb, Croatia; 4 Social and Cognitive Neuroscience Laboratory, Mackenzie Presbyterian University, São Paulo, Brazil; 5 Department of Economics, Norwegian School of Economics, Bergen, Norway; 6 Telenor Research, Oslo, Norway; 7 Macedonian Academy of Sciences and Arts, Skopje, Republic of North Macedonia; 8 Kieskompas - Election Compass, Amsterdam, Netherlands; 9 Department of Political Science & Annenberg School for Communication, University of Pennsylvania, Philadelphia, PA USA; 10 Department of Psychology, Macquarie University, Sydney, NSW Australia; 11 Department of Philosophy, University of St Andrews, St Andrews, Scotland; 12 School of Economics and Finance, University of St Andrews, St Andrews, Scotland; 13 Medicine Faculty, Cooperative University of Colombia, Villavicencio, Colombia; 14 Department of Bioethics, El Bosque University, Bogotá, Colombia; 15 School of Psychology, University of Kent, Canterbury, England; 16 Department of Economics, Middlesex University London, London, England; 17 Darden School of Business, University of Virginia, Charlottesville, VA USA; 18 Questrom School of Business, Boston University, Boston, MA USA; 19 School of Economics and Finance, Queensland University of Technology, Brisbane, QLD Australia; 20 Center for Behavioural Economics, Society and Technology, Queensland University of Technology, Brisbane, QLD Australia; 21 Department of Psychology and Neural Science, New York University, New York, NY USA; 22 Department of Strategy and Management, Norwegian School of Economics, Bergen, Norway; 23 SWPS University of Social Sciences and Humanities, Warsaw, Poland; 24 Department of Psychological Sciences, College of William and Mary, Williamsburg, VA USA; 25 Department of Philosophy, Macquarie University, Sydney, NSW Australia; 26 Stanford Graduate School of Business, Stanford University, Stanford, CA USA; 27 School of Human Sciences, Institute for Lifecourse Development, University of Greenwich, London, England; 28 Department of Experimental Psychology, University of Oxford, Oxford, England; 29 Center for Human Brain Health, School of Psychology, University of Birmingham, Birmingham, England; 30 KU Leuven, Leuven, Belgium; 31 National Research University Higher School of Economics (HSE), Moscow, Russia; 32 De La Salle University, Manila, Philippines; 33 Department of Psychology, University of the Witwatersrand, Johannesburg, South Africa; 34 Department of Political Science, Aarhus University, Aarhus, Denmark; 35 Toronto Metropolitan University, Toronto, Canada; 36 Department of Government, Dartmouth College, Hanover, NH USA; 37 Department of Psychology, University of Toronto, Toronto, ON Canada; 38 Gatton College of Business and Economics, University of Kentucky, Lexington, KY USA; 39 Department of Mass Communication, National University of Science and Technology (NUST), Islamabad, Pakistan; 40 Department of Management, Aarhus University, Aarhus, Denmark; 41 Faculty of Social and Economic Sciences, Comenius University, Bratislava, Slovakia; 42 IntraMed, Buenos Aires, Argentina; 43 University of Colorado Boulder, Boulder, CO USA; 44 ESEIS/COIDESO [ESEIS, Social Studies and Social Intervention Research Center; COIDESO, COIDESO, Center for Research in Contemporary Thought and Innovation for Social Development], University of Huelva, Huelva, Spain; 45 Faculty of Social Work, University of Huelva, Huelva, Spain; 46 WHU - Otto Beisheim School of Management, Vallendar, Germany; 47 Latin American Brain Health Institute (BrainLat), Universidad Adolfo Ibáñez, Santiago, Chile; 48 Cognitive Neuroscience Center (CNC), University of San Andrés, Buenos Aires, Argentina; 49 Global Brain Health Institute (GBHI), University of California San Francisco (UCSF), California, US; & Trinity College Dublin (TCD), Dublin, Ireland; 50 Department of Educational and Counselling Psychology, University of Dhaka, Dhaka, Bangladesh; 51 Department of Psychology, The Hebrew University of Jerusalem, Jerusalem, Israel; 52 Institute of Psychology, Jagiellonian University, Kraków, Poland; 53 Institute of Security and Global Affairs, Leiden University, The Hague, Netherlands; 54 Erasmus School of Law, Erasmus University Rotterdam, Rotterdam, Netherlands; 55 Department of Political Science, Vrije University (VU) Amsterdam, Amsterdam, Netherlands; 56 Department of Digital Humanities, University of Helsinki, Helsinki, Finland; 57 Department of Cognition, Emotion, and Methods in Psychology, University of Vienna, Vienna, Austria; 58 School of Psychology, National Autonomous University of Mexico, Mexico City, Mexico; 59 Department of Economics, National Taiwan University, Taipei, Taiwan; 60 Center for Research in Econometric Theory and Applications, National Taiwan University, Taipei, Taiwan; 61 Tribhuvan University, Kirtipur, Nepal; 62 Department of Leadership and Organizational Behavior, BI Norwegian Business School, Oslo, Norway; 63 Institute of Security and Global Affairs, Leiden University, Leiden, Netherlands; 64 Faculty of Governance and Global Affairs, Leiden University, Leiden, Netherlands; 65 Department of Psychology, University of Limerick, Limerick, Ireland; 66 Department of Psychology, The University of Western Ontario, London, ON Canada; 67 Center for Advanced Hindsight, Duke University, Durham, NC USA; 68 Department of Clinical Neuroscience, Karolinska Institute, Solna, Sweden; 69 Department of Management, University of Agder, Kristiansand, Norway; 70 Institute of Retail Economics, Stockholm, Sweden; 71 Department of Psychology, Lehigh University, Bethlehem, PA USA; 72 Department of Psychology, Monk Prayogshala, Mumbai, India; 73 Faculty of Medicine, Pontifical Javeriana University, Bogotá, Colombia; 74 Department of Management, Technology, and Economics, ETH Zürich, Zürich, Switzerland; 75 Department of Psychiatry and Medical Psychology, Research Institute, Medical University of Plovdiv, Plovdiv, Bulgaria; 76 Humanities and Social Sciences, Indian Institute of Management, Indore, India; 77 Institute of Social Sciences, Belgrade, Serbia; 78 Department of Psychology, Royal Holloway, University of London, London, England; 79 Center for the Politics of Feelings, School of Advanced Study, University of London, London, England; 80 Department of Behavioral and Cognitive Sciences, Faculty of Humanities, Education and Social Sciences, University of Luxembourg, Luxembourg City, Luxembourg; 81 Department of Political Science, National Taiwan University, Taipei, Taiwan; 82 Faculty of Political Science, National School for Political Studies and Public Administration, Bucharest, Romania; 83 Department of Psychology, University of Latvia, Riga, Latvia; 84 Department of Psychology, Princeton University, Princeton, NJ USA; 85 Department of Psychology, Speech, and Hearing, University of Canterbury, Christchurch, New Zealand; 86 Department of Psychology and Neuroscience, Duke University, Durham, NC USA; 87 Department of Psychology, University of Virginia, Charlottesville, VA USA; 88 School of Economics and Management, Hebei University of Technology, Tianjin, PR China; 89 School of Collective Intelligence, Mohammed VI Polytechnic University, Ben Guerir, Morocco; 90 Institute for Research and Development-Kurdistan, Middle East, Iraq; 91 Impact For Development, North Africa, Rabat, Morocco; 92 Department of Psychology, University of Amsterdam, Amsterdam, Netherlands; 93 Department of Psychology, Jose Rizal University, Mandaluyong, Philippines; 94 Department of Philosophy, University Cheikh Anta Diop, Dakar, Senegal; 95 School of Medicine and Health Sciences, University of Rosario, Bogotá, Colombia; 96 Moral Psychology and Decision Sciences Research Incubator, University of Rosario, Bogotá, Colombia; 97 School of Psychological Sciences, University of Melbourne, Parkville, VIC Australia; 98 Department of Psychological and Social Sciences, Penn State Abington, Abington, PA USA; 99 Institute of Psychology, University of Wrocław, Wrocław, Poland; 100 IMT School for Advanced Studies Lucca, Lucca, Italy; 101 Department of Economics and Management, University of Florence, Florence, Italy; 102 Department of Management, London School of Economics and Political Science, London, England; 103 Toulouse Business School, University of Toulouse, Toulouse, France; 104 Social Policy Institute of the Ministry of Labor, Family and Social Affairs of the Slovak Republic, Bratislava, Slovakia; 105 The Institute for Sociology of the Slovak Academy of Sciences, Bratislava, Slovakia; 106 Department of Psychology, Penn State University, University Park, PA USA; 107 Rock Ethics Institute, Penn State University, University Park, PA USA; 108 Department of Psychology, Federal University of Santa Catarina, Florianópolis, Brazil; 109 Department of Economics, University of Bologna, Bologna, Italy; 110 IUSS Cognitive Neuroscience (ICoN) Center, Institute for Advanced Study of Pavia, Pavia, Italy; 111 Cognitive Computational Neuroscience Research Unit, Neurological Institute Foundation Casimiro Mondino, Pavia, Italy; 112 School of Psychology, Shenzhen University, Shenzhen, PR China; 113 Institute for Advanced Study in Toulouse, Université Toulouse 1 Capitole, Toulouse, France; 114 Department of Brain and Behavioral Sciences, University of Pavia, Pavia, Italy; 115 Cracow University of Economics, Kraków, Poland; 116 UBC Sauder School of Business, University of British Columbia, Vancouver, BC Canada; 117 Psychology Department, University of California - Los Angeles, Los Angeles, CA USA; 118 Laboratory of Psychology: Cognition, Behavior, and Communication (LP3C), Rennes 2 University, Rennes, France; 119 Laboratory of Social and Cognitive Psychology, Clermont Auvergne University, CNRS, Clermont-Ferrand, France; 120 Cavite State University-General Trias City Campus, Cavite, Philippines; 121 Crawford School of Public Policy, Australian National University, Canberra, ACT Australia; 122 CESifo, University of Munich, Munich, Germany; 123 Department of International Trade, Boğaziçi University, Istanbul, Turkey; 124 Hult International Business School Dubai, Dubai, UAE; 125 Department of Social Policy and Intervention, University of Oxford, Oxford, England; 126 Department of Media and Communications, University of Sydney, Sydney, NSW Australia; 127 Department of Psychiatry, University of the Witwatersrand, Johannesburg, South Africa; 128 University of Bayreuth, Bayreuth, Germany; 129 Department of Psychology, University of Waterloo, Waterloo, ON Canada; 130 Department of Peace Studies, University of Bradford, Bradford, England; 131 Philosophy and Social Studies Department, Rethymno, Greece; 132 Department of Psychology and Neuroscience, University of North Carolina at Chapel Hill, Chapel Hill, NC USA; 133 Department of Economics, Koc University, Istanbul, Turkey; 134 Department of Media and Communication, City University of Hong Kong, Kowloon Tong, Hong Kong; 135 University of Regensburg, Regensburg, Germany; 136 Graduate School for Transnational Studies, Free University of Berlin, Berlin, Germany; 137 Department of Management and Engineering, Linköping University, Linköping, Sweden; 138 Department of Psychology, University of Koblenz-Landau, Landau, Germany; 139 Department of Psychology, University of Alberta, Edmonton, Canada; 140 LMU Center for Leadership and People Management, Ludwig Maximilian University of Munich, Munich, Germany; 141 Augsburg University for Applied Sciences, Augsburg, Germany; 142 Baruch Ivcher School of Psychology, Reichman University, Herzliya, Israel; 143 Department of Neuroscience and Biomedical Engineering, Aalto University, Espoo, Finland; 144 School of Psychology, Dublin City University, Dublin, Ireland; 145 University of Montana, Missoula, MT USA; 146 Graduate School of Human Sciences, Osaka University, Suita, Japan; 147 SEELE Neuroscience, Mexico City, Mexico; 148 School of Psychology, University of Auckland, Auckland, New Zealand; 149 Faculty of Economics, Maria Curie-Skłodowska University, Lublin, Poland; 150 Department of Psychology, The City University of New York (CUNY) Graduate Center, New York, NY USA; 151 Australian National Centre for the Public Awareness of Science, Australian National University, Canberra, ACT Australia; 152 Department of Social Psychology, Graduate School of Humanities and Sociology, University of Tokyo, Tokyo, Japan; 153 Department of Public Administration and Sociology, Erasmus University Rotterdam, Rotterdam, Netherlands; 154 Center for Social Sciences, Hungarian Academy of Sciences Center of Excellence, Budapest, Hungary; 155 Institute of Psychology, University of Silesia, Katowice, Poland; 156 Complutense University in Madrid, Madrid, Spain; 157 Department of Psychology, University of Belgrade, Belgrade, Serbia; 158 Universidad Nacional de Colombia, Sede de La Paz, La Paz, Colombia; 159 Vidyasagar College For Women, Kolkata, India; 160 AGH University of Science and Technology, Kraków, Poland; 161 Department of Computer Science, University of Colorado Boulder, Boulder, CO USA; 162 Institute of Cognitive Science, University of Colorado Boulder, Boulder, CO USA; 163 Stanford University, Stanford, CA USA; 164 Department of Biology, University of Turku, Turku, Finland; 165 Cooperative University of Colombia, Bogotá, Colombia; 166 Newcastle Business School, University of Newcastle, Callaghan, NSW Australia; 167 Department of Global Economics and Management, University of Groningen, Groningen, Netherlands; 168 Brain and Mind Institute, University of Western Ontario, London, ON Canada; 169 Western Interdisciplinary Research Building, University of Western Ontario, London, ON Canada; 170 Department of Sociology, University of Belgrade, Belgrade, Serbia; 171 Department of Psychology, Carleton University, Ottawa, ON Canada; 172 National University of Political Studies and Public Administration (SNSPA), Bucharest, Romania; 173 Research Institute at Medical University of Plovdiv), Division of Translational Neuroscience, Plovdiv, Bulgaria; 174 Department of Cognitive Science, ENS, EHESS, CNRS, Institut Jean Nicod, PSL Research University, Paris, France; 175 CREMA - Center for Research in Economics, Management and the Arts, Basel, Switzerland; 176 The Warburg Institute, School of Advanced Study, University of London, London, England; 177 Institute of Cognitive Neuroscience, University College London, London, England; 178 Institute for Emerging Market Studies, The Hong Kong University of Science and Technology, Kowloon, Hong Kong; 179 Department of Psychology, Susquehanna University, Selinsgrove, PA USA; 180 Psychology Department, Dokuz Eylül University, İzmir, Turkey; 181 Department of Experimental and Applied Psychology, VU Amsterdam, Amsterdam, Netherlands; 182 Faculty of Design Sciences, University of Antwerp, Antwerpen, Belgium; 183 Department of Behavioural Sciences and Learning (IBL), Linköping University, Linköping, Sweden; 184 Department of Psychology, Education and Child Studies, Erasmus University Rotterdam, Rotterdam, Netherlands; 185 University of Koblenz-Landau, Landau, Germany; 186 Health Communication Research Unit, School of Human and Community Development, University of the Witwatersrand, Johannesburg, South Africa; 187 Department of Entrepreneurship, Innovation, and Technology, Stockholm School of Economics, Stockholm, Sweden; 188 Harvard Business School, Harvard University, Cambridge, MA USA; 189 Nicolaus Copernicus University, Toruń, Poland; 190 University of California, San Diego, La Jolla, CA USA; 191 Kyushu University, Fukuoka, Japan; 192 Department of Psychology, Kadir Has University, Istanbul, Turkey

**Keywords:** Human behaviour, Politics, Decision making

## Abstract

The COVID-19 pandemic has affected all domains of human life, including the economic and social fabric of societies. One of the central strategies for managing public health throughout the pandemic has been through persuasive messaging and collective behaviour change. To help scholars better understand the social and moral psychology behind public health behaviour, we present a dataset comprising of 51,404 individuals from 69 countries. This dataset was collected for the International Collaboration on Social & Moral Psychology of COVID-19 project (ICSMP COVID-19). This social science survey invited participants around the world to complete a series of moral and psychological measures and public health attitudes about COVID-19 during an early phase of the COVID-19 pandemic (between April and June 2020). The survey included seven broad categories of questions: COVID-19 beliefs and compliance behaviours; identity and social attitudes; ideology; health and well-being; moral beliefs and motivation; personality traits; and demographic variables. We report both raw and cleaned data, along with all survey materials, data visualisations, and psychometric evaluations of key variables.

## Background & Summary

Well over two years after the official outbreak^1^, it is evident that the COVID-19 pandemic has affected all domains of human life, including the economic and social fabric of societies^2^ as well as people’s physical and mental health^3^. At the time of writing, the world reached 850 million confirmed infections and up to 18 million deaths^4^. The detrimental effects of the pandemic extend beyond physical health with evidence of increased stress levels^5^ and suicide rates^6^, along with deterioration of general well-being^7^. Such findings reflect the cautionary warnings by Taylor^8^ that the psychological and societal effects are “likely to be more pronounced, more widespread, and longer-lasting than the purely somatic effects of the infection”^8^, p.23.

In the early stages of the pandemic, when vaccines were not yet available, governments introduced non-pharmaceutical interventions to reduce the spread of the SARS-CoV-2 virus^9^. Various contact-restricting policies (e.g., stay-at-home recommendations, curfews, police hours, partial or complete lock-downs) were enacted, and citizens were advised to adhere to public health recommendations (e.g., hand washing, face masks, and spatial distancing). It quickly became clear that behavioural science had a major role to play^10^.

On April 11th, a team of researchers launched a call for international collaboration in social and moral psychology. The initiative quickly gained momentum, gathering a consortium of over 250 academics worldwide. The aim of this project was to collect data from as many countries as possible to serve as a public good for the scientific community by allowing future research to draw on this broad database collected during this early phase of the COVID-19 pandemic. The survey, developed by the initial team, was circulated among the national teams, who provided feedback, translated it into 32 languages, and disseminated it online. The project concluded with responses from a total of 51,404 participants from 69 countries, 77 samples, between April 22nd and June 3rd, 2020.

A key goal of the project was to test the hypothesis that national identity predicts support for public health measures during the COVID-19 pandemic, which has since been confirmed^11,12^. In addition to collecting variables to test this hypothesis, we collected data on a variety of other social and moral constructs to make of our multi-country large-scale survey a rich resource for future research. The survey focused on the following areas: on a) COVID-19 beliefs and compliance behaviours (COVID-19 public health support, COVID-19 risk perception, COVID-19 conspiracy beliefs, and COVID-19 testing behaviour); b) identity and social attitudes (national identification, national narcissism, and social belonging); c) ideology (political ideology); d) health and well-being (subjective physical health, a wealth ladder ranking, and psychological well-being); e) moral beliefs and motivation (generosity, morality as cooperation, moral identity, and moral circle); f) personality traits and dispositions (open-mindedness, self-esteem, trait optimism, trait self-control, narcissism, and cognitive reflection); and g) demographic variables (i.e., sex, age, marital status, number of children, and employment status).

Using this dataset, project team members have pre-registered a variety of secondary hypotheses (see icsmp-covid19.netlify.app/preregistration), several of which have already been published^11–23^. In this paper, we present the complete ICSMP datasets to facilitate its findability, accessibility, interoperability, and reuse (FAIR;^24,25^) and maximize its educational impact^26–28^.

## Methods

When possible, we used articles published in Nature Scientific Data presenting social sciences data as blueprints^5,29^. Given the urgent call for COVID-19 research, this study received an umbrella ethical approval from the University of Kent (see osf.io/ce638) but also complied with local ethics, norms, and regulations in the countries where the data were collected.

### Participants

**Fig. 1:**
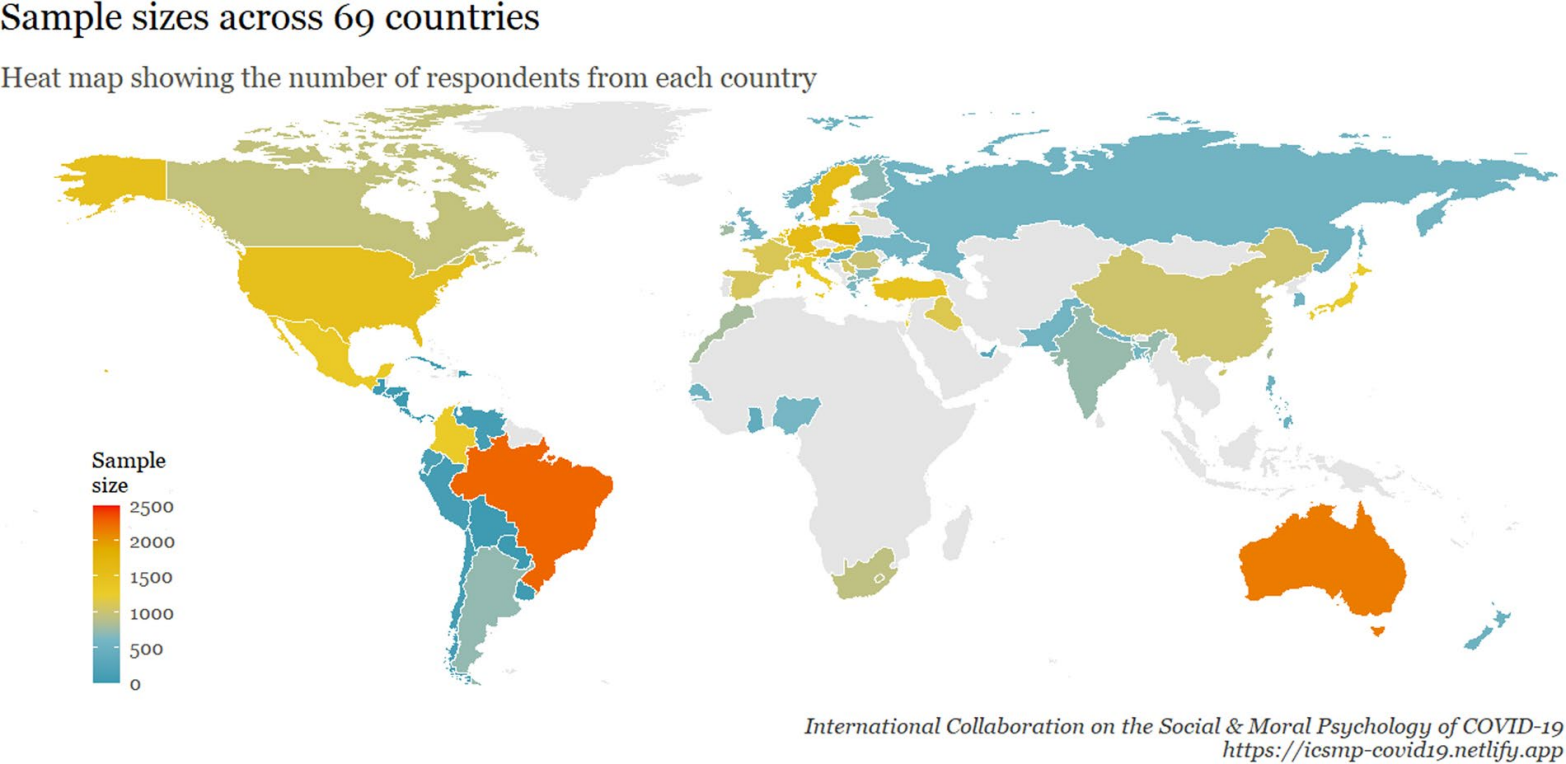
A world map visualizing the number of participants in each surveyed country. ***Note:*** This heat map shows the number of respondents from each country. The gray areas are the countries that are not covered by the data, and the colour scale shows the size of the sample in accordance with the scale on the lower left side.

**Fig. 2:**
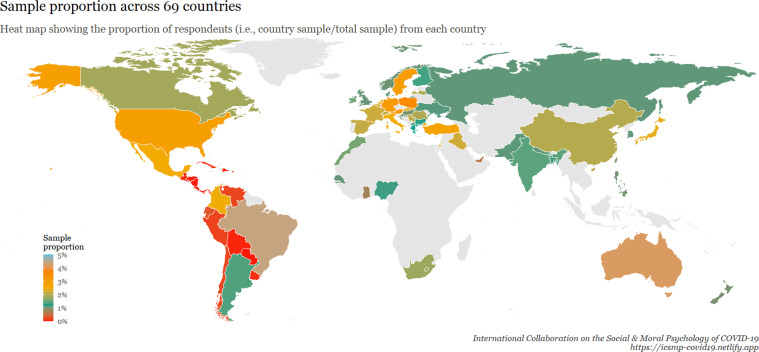
International Collaboration on the Social and Moral Psychology of COVID-19: Investigated constructs, items and variables.

**Fig. 3:**
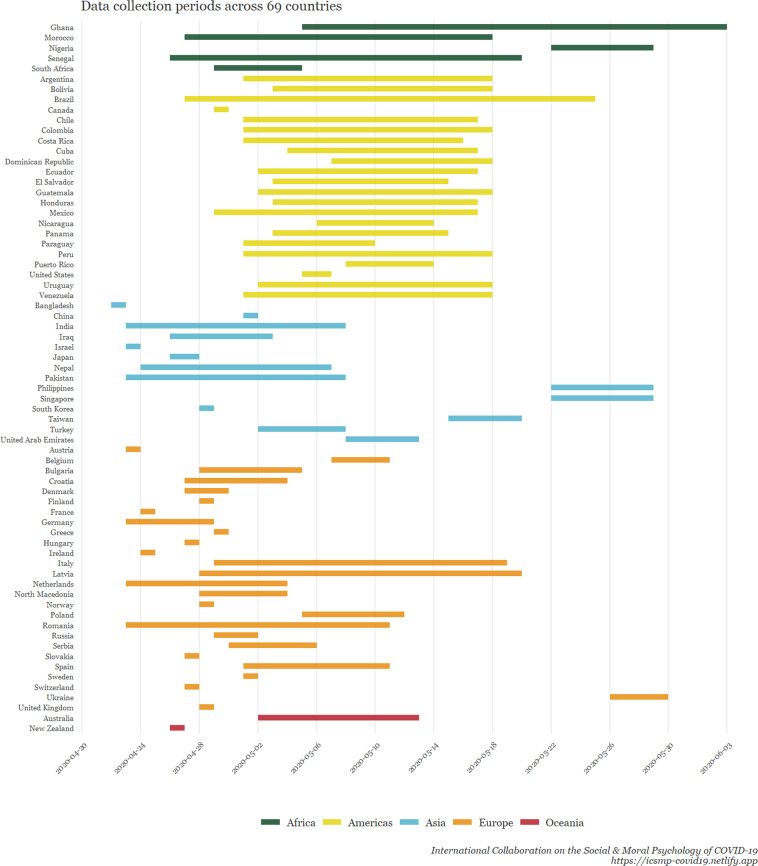
Gantt Chart illustrating the data collection periods for each surveyed country.

A total of 51,404 individuals from 77 samples across 69 countries participated in our survey. The inclusion criteria were the following: being 18 years of age and older, and giving informed consent (although researchers were encouraged to, ideally, recruit representative samples regarding age and gender). Data were collected between April 22nd and June 3rd, 2020. Figure 1 displays *where* the data were collected, coloured according to national sample size. Figure 2 displays the proportion of respondents in relation to the full sample. Figure 3 shows *when* the data were collected in each country.

Demographic variables across countries are summarised in several tables: Tables 1, 2 show the number of participants, the mean proportion of non-missing ‘valid’ answers, and age. Tables 3, 4 illustrate the distribution of gender; Tables 5, 6 show employment status; and Tables 7–9 show marital status and number of children. When multiple samples were collected within the same country, data were split into numbered subgroups (e.g., for Brazil, which has three samples, they were flagged as Brazil_1, Brazil_2 and Brazil_3). Note that in the tables above, we kept country subsamples separated to highlight they were collected by different teams, often using different sampling methodologies or languages, which impact their characteristics (e.g., representativeness).

**Table 1:** Sample size, average proportion of valid answers, age of respondents and the number of data collections in 69 countries (A-M).

Sample	Country	N	*% Valid Answers*		*Age*		Multiple datasets
			<50%	<90%	*μ*_*Age*_	*sd*_*Age*_	per country
AR	Argentina	721	1.00	1.00	47.38	15.29	1
AU	Australia	2161	1.00	1.00	46.92	17.59	1
AT	Austria	1605	0.90	0.87	49.77	14.13	1
BD	Bangladesh	596	0.82	0.67	31.90	10.89	1
BE	Belgium	1159	1.00	1.00	46.29	18.67	1
BO	Bolivia	29	1.00	1.00	43.41	12.98	1
BR_1	Brazil_1	961	0.99	0.99	39.31	14.57	3
BR_2	Brazil_2	1301	0.75	0.67	34.89	13.12	3
BR_3	Brazil_3	6	1.00	1.00	40.33	13.14	3
BG	Bulgaria	666	1.00	0.96	30.69	11.13	1
CA_e	Canada_english	792	1.00	1.00	42.70	17.39	2
CA_f	Canada_french	171	1.00	1.00	46.83	16.97	2
CL	Chile	97	1.00	1.00	49.21	15.47	1
CN	China	1030	1.00	1.00	43.24	14.02	1
CO_1	Colombia_1	731	0.99	0.91	37.26	14.68	2
CO_2	Colombia_2	546	1.00	1.00	44.91	15.16	2
CR	Costa Rica	25	1.00	1.00	44.64	12.73	1
HR	Croatia	515	1.00	1.00	45.91	14.56	1
CU	Cuba	43	1.00	1.00	48.65	12.73	1
DK	Denmark	566	1.00	1.00	48.69	17.54	1
DO	Dominican Republic	36	1.00	1.00	40.39	12.46	1
EC	Ecuador	148	1.00	1.00	40.63	11.98	1
SV	El Salvador	28	1.00	1.00	46.43	11.51	1
FI	Finland	698	0.99	0.98	38.64	13.77	1
FR	France	1119	1.00	0.99	43.18	16.20	1
DE	Germany	1587	1.00	1.00	49.58	16.14	1
GH	Ghana	390	0.68	0.49	31.46	7.54	1
GR	Greece	640	1.00	1.00	29.77	11.43	1
GT	Guatemala	48	1.00	1.00	44.67	13.31	1
HN	Honduras	24	1.00	1.00	39.25	14.30	1
HU	Hungary	506	1.00	1.00	48.53	16.54	1
IN_1	India_1	312	0.87	0.81	26.94	8.49	2
IN_2	India_2	429	0.94	0.84	36.81	12.05	2
IQ	Iraq	1142	0.57	0.48	31.03	14.13	1
IE	Ireland	785	0.96	0.95	38.23	14.63	1
IL	Israel	1253	1.00	1.00	41.13	15.25	1
IT_1	Italy_1	998	0.99	0.99	46.41	16.26	2
IT_2	Italy_2	284	1.00	1.00	47.35	18.07	2
JP	Japan	1239	0.96	0.93	47.10	15.21	1
KR	Korea	555	0.92	0.89	41.83	13.90	1
LV	Latvia	1008	1.00	1.00	45.60	14.11	1
MK	Macedonia	726	0.97	0.96	38.13	11.63	1
MX_1	Mexico_1	804	0.94	0.93	47.81	13.89	2
MX_2	Mexico_2	507	1.00	1.00	47.77	13.54	2
MA	Morocco	812	0.81	0.71	31.95	12.27	1

***Note:*** Country = country names in accordance with ISO3 codes, N = number of respondents in each country. <50% and <90% = average proportion of valid (non NA) answers that are below 0.5 and 0.9 respectively in the subject level. *μ*_*Age*_ = mean age and *sd*_*Age*_ = standard deviation of the age, Multiple datasets = whether there were multiple data collections in the country. Tables 1, 2 show the number of participants, the mean proportion of non-missing ‘valid’ answers, and age. When multiple samples were collected within the same country, data were split into numbered subgroups (e.g., for Brazil, which has three samples, they were flagged as Brazil_1, Brazil_2 and Brazil_3). Multiple subsamples can be observed for Brazil, Canada, Colombia, India, Italy, Mexico and Romania. Note that in all the tables, we kept country subsamples separated to highlight they were collected by different teams, often using different sampling methodologies or languages, which impact their characteristics (e.g., representativeness).

**Table 2:** Sample size, average proportion of valid answers, age of respondents and the number of data collections in 69 countries (N-V).

Sample	Country	N	*% Valid Answers*		*Age*		Multiple datasets
			<50%	<90%	*μ*_*Age*_	*sd*_*Age*_	per country
NP	Nepal	563	0.78	0.61	28.06	7.58	1
NL	Netherlands	1297	1.00	0.99	49.63	16.83	1
NZ	New Zealand	510	1.00	1.00	45.76	17.62	1
NI	Nicaragua	16	1.00	1.00	42.75	14.84	1
NG	Nigeria	608	0.93	0.87	32.08	10.81	1
NO	Norway	532	1.00	1.00	47.04	17.39	1
PK	Pakistan	565	0.90	0.85	26.94	8.38	1
PA	Panama	18	1.00	1.00	44.11	17.32	1
PY	Paraguay	16	1.00	1.00	38.94	9.33	1
PE	Peru	91	1.00	1.00	46.21	14.44	1
PH	Philippines	524	0.98	0.96	36.74	12.27	1
PL	Poland	1817	1.00	1.00	46.44	17.09	1
PR	Puerto Rico	2	1.00	1.00	64.00	16.97	1
RO_1	Romania_1	500	1.00	1.00	42.26	13.45	2
RO_2	Romania_2	505	1.00	0.99	42.53	14.50	2
RU	Russian Federation	558	1.00	1.00	45.02	15.46	1
SN	Senegal	552	0.62	0.51	34.36	12.43	1
RS	Serbia	1070	0.88	0.71	42.92	11.93	1
SG	Singapore	564	0.96	0.92	43.06	13.73	1
SK	Slovakia	1265	1.00	1.00	44.19	15.88	1
ZA	South Africa	939	0.82	0.56	39.90	13.44	1
ES	Spain	1090	1.00	0.99	46.01	13.68	1
SE	Sweden	1568	1.00	1.00	52.90	15.42	1
CH	Switzerland	1056	1.00	1.00	47.94	16.66	1
TW	Taiwan	833	1.00	1.00	43.99	13.25	1
TR	Turkey	1455	1.00	0.99	37.23	15.24	1
UA	Ukraine	577	1.00	1.00	37.45	8.03	1
AE	United Arab Emirates	313	0.71	0.59	31.77	8.59	1
GB	United Kingdom	550	1.00	1.00	45.66	15.62	1
US	United States of America	1506	1.00	0.99	44.23	16.60	1
UY	Uruguay	49	1.00	1.00	52.88	13.70	1
VE	Venezuela	96	1.00	1.00	46.53	12.97	1

***Note:*** Country = country names in accordance with ISO3 codes, N = number of respondents in each country. <50% and <90% = average proportion of valid (non NA) answers that are below 0.5 and 0.9 respectively in the subject level. *μ*_*Age*_ = mean age and *sd*_*Age*_ = standard deviation of the age, Multiple datasets = whether there were multiple data collections in the country. Tables 1, 2 show the number of participants, the mean proportion of non-missing ‘valid’ answers, and age. When multiple samples were collected within the same country, data were split into numbered subgroups (e.g., for Brazil, which has three samples, they were flagged as Brazil_1, Brazil_2 and Brazil_3). Multiple subsamples can be observed for Brazil, Canada, Colombia, India, Italy, Mexico and Romania. Note that in all the tables, we kept country subsamples separated to highlight they were collected by different teams, often using different sampling methodologies or languages, which impact their characteristics (e.g., representativeness).

**Table 3:** Distribution of sex in 69 countries (A-M).

Country	% Female	% Male	% Other	% Unreported
Argentina	0.69	0.31	0.00	0.00
Australia	0.51	0.48	0.01	0.00
Austria	0.46	0.41	0.00	0.13
Bangladesh	0.37	0.31	0.01	0.31
Belgium	0.41	0.59	0.00	0.00
Bolivia	0.59	0.41	0.00	0.00
Brazil_1	0.49	0.50	0.01	0.01
Brazil_2	0.47	0.19	0.00	0.33
Brazil_3	0.83	0.17	0.00	0.00
Bulgaria	0.65	0.34	0.00	0.01
Canada_English	0.62	0.38	0.01	0.00
Canada_French	0.54	0.46	0.00	0.00
Chile	0.65	0.35	0.00	0.00
China	0.49	0.51	0.00	0.00
Colombia_1	0.62	0.37	0.00	0.01
Colombia_2	0.63	0.37	0.00	0.00
Costa Rica	0.36	0.64	0.00	0.00
Croatia	0.52	0.48	0.00	0.01
Cuba	0.51	0.49	0.00	0.00
Denmark	0.49	0.51	0.00	0.00
Dominican Republic	0.81	0.19	0.00	0.00
Ecuador	0.55	0.45	0.00	0.00
El Salvador	0.54	0.46	0.00	0.00
Finland	0.45	0.48	0.05	0.02
France	0.55	0.45	0.00	0.00
Germany	0.50	0.50	0.00	0.00
Ghana	0.26	0.53	0.00	0.22
Greece	0.35	0.65	0.00	0.00
Guatemala	0.44	0.56	0.00	0.00
Honduras	0.71	0.29	0.00	0.00
Hungary	0.52	0.48	0.00	0.00
India_1	0.42	0.38	0.02	0.18
India_2	0.31	0.59	0.01	0.10
Iraq	0.23	0.26	0.01	0.50
Ireland	0.63	0.31	0.00	0.05
Israel	0.51	0.49	0.00	0.00
Italy_1	0.50	0.49	0.00	0.00
Italy_2	0.66	0.33	0.00	0.01
Japan	0.48	0.46	0.00	0.06
Korea	0.42	0.48	0.00	0.10
Latvia	0.63	0.37	0.00	0.00
Macedonia	0.54	0.43	0.01	0.03
Mexico_1	0.39	0.53	0.00	0.07
Mexico_2	0.61	0.38	0.00	0.00
Morocco	0.52	0.47	0.01	0.00

*Note:* Country = country names in accordance with ISO3 codes, % Female = Proportion of female respondents in the country, % Male = proportion of male respondents, % Other = proportion of non-binary respondents and % NA = proportion of the unreported sex.

**Table 4:** Distribution of sex in 69 countries (N-V).

Country	% Female	% Male	% Other	% Unreported
Nepal	0.33	0.29	0.01	0.37
Netherlands	0.46	0.54	0.00	0.00
New Zealand	0.50	0.50	0.00	0.00
Nicaragua	0.62	0.38	0.00	0.00
Nigeria	0.49	0.51	0.00	0.00
Norway	0.53	0.46	0.00	0.00
Pakistan	0.46	0.40	0.00	0.14
Panama	0.67	0.33	0.00	0.00
Paraguay	0.88	0.12	0.00	0.00
Peru	0.45	0.55	0.00	0.00
Philippines	0.50	0.50	0.00	0.00
Poland	0.49	0.50	0.00	0.00
Puerto Rico	0.50	0.50	0.00	0.00
Romania_1	0.52	0.48	0.00	0.00
Romania_2	0.49	0.50	0.00	0.00
Russian Federation	0.53	0.47	0.00	0.00
Senegal	0.37	0.63	0.01	0.00
Serbia	0.53	0.19	0.00	0.28
Singapore	0.51	0.49	0.00	0.00
Slovakia	0.50	0.50	0.00	0.00
South Africa	0.51	0.17	0.00	0.31
Spain	0.33	0.67	0.00	0.00
Sweden	0.40	0.59	0.00	0.00
Switzerland	0.51	0.49	0.00	0.00
Taiwan	0.50	0.50	0.00	0.00
Turkey	0.51	0.49	0.00	0.00
Ukraine	0.52	0.47	0.00	0.00
United Arab Emirates	0.29	0.31	0.00	0.40
United Kingdom	0.51	0.49	0.00	0.00
United States of America	0.51	0.48	0.00	0.00
Uruguay	0.69	0.31	0.00	0.00
Venezuela	0.56	0.44	0.00	0.00

*Note:* Country = country names in accordance with ISO3 codes, % Female = Proportion of female respondents in the country, % Male = proportion of male respondents, % Other = proportion of non-binary respondents and % NA = proportion of the unreported sex.

**Table 5:** Distribution of employment status in 69 countries (A-M).

Country	% Full	% Part	% Unemp.	% Student	% Retired	% Other	% Unreported
Argentina	0.45	0.15	0.02	0.08	0.08	0.22	0.00
Australia	0.36	0.18	0.11	0.05	0.23	0.07	0.00
Austria	0.36	0.13	0.02	0.05	0.12	0.20	0.13
Bangladesh	0.18	0.15	0.08	0.21	0.02	0.04	0.32
Belgium	0.28	0.04	0.03	0.25	0.25	0.14	0.00
Bolivia	0.52	0.14	0.07	0.07	0.00	0.21	0.00
Brazil_1	0.51	0.10	0.11	0.09	0.09	0.09	0.01
Brazil_2	0.25	0.08	0.06	0.16	0.04	0.08	0.33
Brazil_3	0.50	0.00	0.00	0.33	0.00	0.17	0.00
Bulgaria	0.37	0.06	0.06	0.24	0.01	0.23	0.03
Canada_English	0.41	0.12	0.09	0.11	0.18	0.09	0.00
Canada_French	0.00	0.00	0.63	0.05	0.25	0.08	0.00
Chile	0.40	0.16	0.04	0.04	0.07	0.28	0.00
China	0.73	0.01	0.01	0.05	0.20	0.00	0.00
Colombia_1	0.42	0.07	0.09	0.26	0.05	0.11	0.02
Colombia_2	0.40	0.15	0.04	0.12	0.07	0.22	0.00
Costa Rica	0.68	0.04	0.12	0.00	0.08	0.08	0.00
Croatia	0.48	0.03	0.16	0.05	0.24	0.05	0.00
Cuba	0.74	0.07	0.09	0.02	0.02	0.05	0.00
Denmark	0.41	0.07	0.07	0.10	0.29	0.07	0.00
Dominican Republic	0.56	0.14	0.08	0.11	0.03	0.08	0.00
Ecuador	0.57	0.10	0.06	0.07	0.05	0.14	0.00
El Salvador	0.68	0.07	0.07	0.04	0.00	0.14	0.00
Finland	0.44	0.08	0.09	0.19	0.08	0.10	0.02
France	0.55	0.07	0.07	0.08	0.18	0.05	0.00
Germany	0.37	0.13	0.05	0.07	0.29	0.09	0.00
Ghana	0.31	0.08	0.11	0.22	0.01	0.05	0.22
Greece	0.33	0.10	0.14	0.37	0.03	0.03	0.00
Guatemala	0.56	0.08	0.04	0.04	0.04	0.23	0.00
Honduras	0.46	0.38	0.08	0.04	0.00	0.04	0.00
Hungary	0.44	0.07	0.07	0.05	0.29	0.07	0.00
India_1	0.31	0.05	0.06	0.33	0.01	0.05	0.18
India_2	0.37	0.11	0.09	0.10	0.05	0.19	0.10
Iraq	0.09	0.08	0.09	0.17	0.01	0.04	0.50
Ireland	0.42	0.12	0.05	0.18	0.06	0.12	0.05
Israel	0.39	0.13	0.15	0.06	0.09	0.18	0.00
Italy_1	0.42	0.12	0.13	0.08	0.17	0.08	0.00
Italy_2	0.37	0.07	0.04	0.15	0.25	0.11	0.00
Japan	0.44	0.12	0.16	0.05	0.10	0.06	0.06
Korea	0.49	0.12	0.06	0.08	0.06	0.09	0.10
Latvia	0.63	0.08	0.06	0.07	0.10	0.08	0.00
Macedonia	0.70	0.04	0.07	0.08	0.02	0.06	0.03
Mexico_1	0.45	0.12	0.08	0.03	0.10	0.16	0.07
Mexico_2	0.52	0.15	0.03	0.05	0.07	0.18	0.00
Morocco	0.38	0.09	0.12	0.29	0.03	0.09	0.01

*Note:* Country = country names in accordance with ISO3 codes, % Full = Proportion of full time workers, % Part = proportion of part time workers, % Unemp. = proportion of unemployed respondents, % Student = proportion of students, % Retired = proportion of retirees, % Other = proportion of respondents who do not fit in the mentioned categories and % NA = proportion of the unreported employment status.

**Table 6:** Distribution of employment status in 69 countries (N-V).

Country	% Full	% Part	% Unemp.	% Student	% Retired	% Other	% Unreported
Nepal	0.25	0.08	0.07	0.19	0.01	0.04	0.37
Netherlands	0.31	0.17	0.04	0.08	0.20	0.19	0.00
New Zealand	0.40	0.16	0.10	0.05	0.16	0.12	0.00
Nicaragua	0.44	0.25	0.06	0.00	0.06	0.19	0.00
Nigeria	0.30	0.14	0.17	0.18	0.01	0.06	0.13
Norway	0.45	0.09	0.03	0.06	0.20	0.15	0.00
Pakistan	0.24	0.05	0.07	0.43	0.01	0.06	0.14
Panama	0.50	0.00	0.11	0.06	0.11	0.22	0.00
Paraguay	0.62	0.38	0.00	0.00	0.00	0.00	0.00
Peru	0.49	0.21	0.07	0.08	0.07	0.09	0.00
Philippines	0.47	0.12	0.15	0.09	0.03	0.11	0.03
Poland	0.37	0.07	0.13	0.07	0.26	0.10	0.00
Puerto Rico	0.50	0.00	0.00	0.00	0.00	0.50	0.00
Romania_1	0.63	0.04	0.08	0.07	0.13	0.05	0.00
Romania_2	0.58	0.05	0.08	0.08	0.14	0.08	0.00
Russian Federation	0.26	0.20	0.23	0.05	0.24	0.02	0.00
Senegal	0.51	0.05	0.06	0.23	0.01	0.13	0.00
Serbia	0.49	0.03	0.06	0.05	0.05	0.00	0.33
Singapore	0.63	0.06	0.08	0.04	0.05	0.05	0.07
Slovakia	0.48	0.05	0.08	0.07	0.24	0.08	0.00
South Africa	0.39	0.06	0.04	0.05	0.04	0.10	0.31
Spain	0.54	0.07	0.09	0.05	0.13	0.11	0.00
Sweden	0.51	0.06	0.03	0.05	0.27	0.09	0.00
Switzerland	0.37	0.18	0.06	0.07	0.20	0.11	0.00
Taiwan	0.57	0.10	0.06	0.07	0.14	0.07	0.00
Turkey	0.37	0.07	0.11	0.20	0.10	0.16	0.00
Ukraine	0.61	0.14	0.12	0.02	0.02	0.09	0.00
United Arab Emirates	0.30	0.02	0.05	0.18	0.00	0.05	0.40
United Kingdom	0.40	0.17	0.11	0.05	0.17	0.10	0.00
United States of America	0.48	0.11	0.12	0.04	0.18	0.06	0.00
Uruguay	0.53	0.14	0.00	0.06	0.12	0.14	0.00
Venezuela	0.46	0.12	0.07	0.02	0.03	0.29	0.00

*Note:* Country = country names in accordance with ISO3 codes, % Full = Proportion of full time workers, % Part = proportion of part time workers, % Unemp. = proportion of unemployed respondents, % Student = proportion of students, % Retired = proportion of retirees, % Other = proportion of respondents who do not fit in the mentioned categories and % NA = proportion of the unreported employment status.

**Table 7:** Distribution of marital status and number of children in 69 countries (A-H).

Country	Marital Status				Number of Children						
	Single	Relation	Married	Unreported (MS)	0	1	2	3	4	≥4	Unreported (Child.)
Argentina	0.29	0.27	0.44	0.00	0.37	0.16	0.26	0.14	0.05	0.02	0.00
Australia	0.37	0.15	0.48	0.00	0.44	0.15	0.24	0.10	0.04	0.02	0.00
Austria	0.20	0.24	0.43	0.13	0.32	0.17	0.23	0.11	0.03	0.01	0.13
Bangladesh	0.33	0.04	0.31	0.32	0.36	0.07	0.10	0.03	0.00	0.01	0.43
Belgium	0.37	0.26	0.36	0.00	0.57	0.12	0.19	0.08	0.03	0.01	0.00
Bolivia	0.38	0.10	0.52	0.00	0.41	0.14	0.28	0.10	0.07	0.00	0.00
Brazil_1	0.40	0.14	0.45	0.01	0.44	0.23	0.20	0.09	0.02	0.01	0.01
Brazil_2	0.24	0.21	0.22	0.33	0.45	0.10	0.09	0.03	0.00	0.00	0.33
Brazil_3	0.17	0.33	0.50	0.00	0.67	0.33	0.00	0.00	0.00	0.00	0.00
Bulgaria	0.40	0.37	0.21	0.02	0.67	0.16	0.13	0.01	0.00	0.01	0.02
Canada_English	0.40	0.21	0.39	0.00	0.57	0.15	0.16	0.08	0.03	0.01	0.00
Canada_French	0.49	0.22	0.29	0.00	0.58	0.16	0.17	0.06	0.02	0.01	0.00
Chile	0.38	0.18	0.44	0.00	0.32	0.12	0.27	0.20	0.07	0.02	0.00
China	0.11	0.05	0.84	0.00	0.20	0.74	0.06	0.00	0.00	0.00	0.00
Colombia_1	0.40	0.29	0.30	0.01	0.55	0.16	0.18	0.07	0.02	0.01	0.02
Colombia_2	0.32	0.21	0.47	0.00	0.39	0.16	0.27	0.12	0.03	0.02	0.00
Costa Rica	0.44	0.12	0.44	0.00	0.56	0.08	0.04	0.12	0.16	0.04	0.00
Croatia	0.21	0.14	0.61	0.04	0.34	0.17	0.34	0.10	0.02	0.02	0.01
Cuba	0.23	0.21	0.56	0.00	0.19	0.40	0.23	0.12	0.05	0.02	0.00
Denmark	0.28	0.26	0.46	0.00	0.39	0.16	0.31	0.09	0.02	0.02	0.00
Dominican Republic	0.44	0.25	0.31	0.00	0.50	0.28	0.14	0.08	0.00	0.00	0.00
Ecuador	0.36	0.16	0.48	0.00	0.43	0.18	0.26	0.09	0.03	0.03	0.00
El Salvador	0.39	0.11	0.50	0.00	0.36	0.25	0.21	0.14	0.00	0.04	0.00
Finland	0.37	0.34	0.27	0.02	0.63	0.11	0.15	0.05	0.02	0.03	0.02
France	0.35	0.30	0.35	0.00	0.58	0.20	0.15	0.05	0.01	0.01	0.00
Germany	0.38	0.19	0.43	0.00	0.48	0.19	0.23	0.08	0.01	0.01	0.00
Ghana	0.36	0.11	0.32	0.21	0.00	0.00	0.00	0.00	0.00	0.00	1.00
Greece	0.45	0.38	0.16	0.00	0.86	0.07	0.06	0.01	0.00	0.00	0.00
Guatemala	0.29	0.25	0.46	0.00	0.40	0.17	0.25	0.10	0.06	0.02	0.00
Honduras	0.38	0.17	0.46	0.00	0.58	0.12	0.00	0.04	0.17	0.08	0.00
Hungary	0.30	0.27	0.43	0.00	0.39	0.25	0.26	0.08	0.01	0.01	0.00

*Note:* Country = country names in accordance with ISO3 codes, Columns 2-5 shows the proportion of different marital status, NA(MS) = unreported marital status, Columns 6-12 shows proportion of respondents by the number of children they have and NA(Child.) = proportion of unreported number of children.

**Table 8:** Distribution of marital status and number of children in 69 countries (I-R).

Country	Marital Status				Number of Children						
	Single	Relation	Married	Unreported (MS)	0	1	2	3	4	≥4	Unreported (Child.)
India_1	0.55	0.14	0.13	0.18	0.69	0.03	0.07	0.00	0.00	0.00	0.20
India_2	0.29	0.07	0.55	0.10	0.20	0.29	0.18	0.01	0.00	0.00	0.31
Iraq	0.26	0.04	0.20	0.50	0.30	0.03	0.05	0.04	0.03	0.03	0.52
Ireland	0.32	0.28	0.34	0.05	0.52	0.10	0.17	0.09	0.05	0.02	0.06
Israel	0.24	0.11	0.55	0.09	0.38	0.12	0.20	0.18	0.06	0.05	0.00
Italy_1	0.26	0.25	0.49	0.00	0.44	0.25	0.25	0.05	0.01	0.00	0.00
Italy_2	0.23	0.30	0.46	0.00	0.49	0.20	0.25	0.06	0.00	0.00	0.00
Japan	0.35	0.05	0.54	0.06	0.46	0.14	0.23	0.08	0.02	0.00	0.07
Korea	0.35	0.07	0.49	0.10	0.44	0.16	0.25	0.03	0.01	0.00	0.10
Latvia	0.34	0.25	0.42	0.00	0.00	0.32	0.19	0.31	0.12	0.05	0.00
Macedonia	0.30	0.19	0.48	0.03	0.50	0.17	0.26	0.04	0.00	0.00	0.04
Mexico_1	0.26	0.18	0.49	0.07	0.34	0.13	0.25	0.14	0.04	0.03	0.07
Mexico_2	0.31	0.19	0.50	0.00	0.29	0.18	0.32	0.15	0.04	0.02	0.00
Morocco	0.57	0.09	0.33	0.01	0.70	0.09	0.10	0.06	0.01	0.01	0.02
Nepal	0.36	0.05	0.21	0.37	0.46	0.08	0.06	0.01	0.00	0.00	0.39
Netherlands	0.29	0.27	0.43	0.00	0.41	0.12	0.29	0.13	0.03	0.02	0.00
New Zealand	0.39	0.20	0.41	0.00	0.41	0.16	0.21	0.13	0.06	0.04	0.00
Nicaragua	0.19	0.25	0.56	0.00	0.25	0.12	0.25	0.19	0.12	0.06	0.00
Nigeria	0.42	0.11	0.34	0.13	0.51	0.10	0.12	0.08	0.03	0.02	0.13
Norway	0.32	0.26	0.42	0.00	0.41	0.15	0.24	0.16	0.03	0.01	0.00
Pakistan	0.51	0.10	0.24	0.14	0.66	0.07	0.07	0.02	0.01	0.01	0.15
Panama	0.33	0.17	0.50	0.00	0.44	0.11	0.28	0.11	0.00	0.06	0.00
Paraguay	0.56	0.31	0.12	0.00	0.44	0.06	0.31	0.06	0.12	0.00	0.00
Peru	0.40	0.14	0.46	0.00	0.35	0.20	0.29	0.13	0.01	0.02	0.00
Philippines	0.44	0.15	0.38	0.03	0.46	0.21	0.17	0.09	0.02	0.02	0.03
Poland	0.29	0.21	0.50	0.00	0.33	0.22	0.31	0.10	0.03	0.01	0.00
Puerto Rico	0.00	0.50	0.50	0.00	0.00	0.00	0.00	0.50	0.50	0.00	0.00
Romania_1	0.32	0.13	0.55	0.00	0.65	0.22	0.11	0.01	0.00	0.00	0.00
Romania_2	0.27	0.19	0.54	0.00	0.40	0.32	0.22	0.04	0.01	0.01	0.00
Russian Federation	0.41	0.15	0.44	0.00	0.39	0.28	0.26	0.05	0.01	0.01	0.00

*Note:* Country = country names in accordance with ISO3 codes, Columns 2-5 shows the proportion of different marital status, NA(MS) = unreported marital status, Columns 6-12 shows proportion of respondents by the number of children they have and NA(Child.) = proportion of unreported number of children.

**Table 9:** Distribution of marital status and number of children in 69 countries (S-V).

Country	Marital Status				Number of Children						
	Single	Relation	Married	Unreported (MS)	0	1	2	3	4	≥4	Unreported (Child.)
Senegal	0.48	0.08	0.44	0.00	0.50	0.13	0.11	0.12	0.06	0.07	0.01
Serbia	0.19	0.15	0.38	0.28	0.28	0.16	0.21	0.05	0.01	0.01	0.29
Singapore	0.31	0.08	0.53	0.07	0.44	0.18	0.21	0.08	0.01	0.01	0.07
Slovakia	0.28	0.25	0.47	0.00	0.37	0.18	0.31	0.11	0.03	0.01	0.00
South Africa	0.23	0.16	0.30	0.32	0.32	0.11	0.16	0.07	0.01	0.01	0.32
Spain	0.24	0.27	0.49	0.00	0.49	0.17	0.27	0.06	0.01	0.00	0.00
Sweden	0.27	0.27	0.46	0.00	0.29	0.14	0.33	0.16	0.05	0.03	0.00
Switzerland	0.31	0.28	0.41	0.00	0.46	0.19	0.25	0.08	0.03	0.01	0.00
Taiwan	0.35	0.11	0.54	0.00	0.46	0.16	0.27	0.09	0.02	0.00	0.00
Turkey	0.37	0.06	0.57	0.00	0.42	0.12	0.26	0.11	0.03	0.05	0.00
Ukraine	0.19	0.12	0.62	0.07	0.33	0.39	0.24	0.03	0.01	0.00	0.00
United Arab Emirates	0.26	0.15	0.20	0.40	0.40	0.08	0.07	0.04	0.00	0.01	0.40
United Kingdom	0.33	0.24	0.42	0.00	0.50	0.15	0.24	0.07	0.03	0.01	0.00
United States of America	0.41	0.11	0.48	0.00	0.47	0.18	0.23	0.08	0.02	0.02	0.00
Uruguay	0.29	0.14	0.57	0.00	0.27	0.22	0.37	0.08	0.06	0.00	0.00
Venezuela	0.28	0.23	0.49	0.00	0.31	0.19	0.30	0.15	0.05	0.00	0.00

*Note:* Country = country names in accordance with ISO3 codes, Columns 2-5 shows the proportion of different marital status, NA(MS) = unreported marital status, Columns 6-12 shows proportion of respondents by the number of children they have and NA(Child.) = proportion of unreported number of children.

For the most part, participants were recruited via professional survey research companies and were incentivised to participate. In countries that, to our knowledge, did not possess polling infrastructure^30^, incentivising participants was not feasible. To collect data in these countries, leaders of national teams relied on online volunteers recruited via media appeals, mailing lists, advertisements on news aggregators, local communities and bloggers, and private messaging apps such as WhatsApp or WeChat.

### Materials

**Fig. 4:**
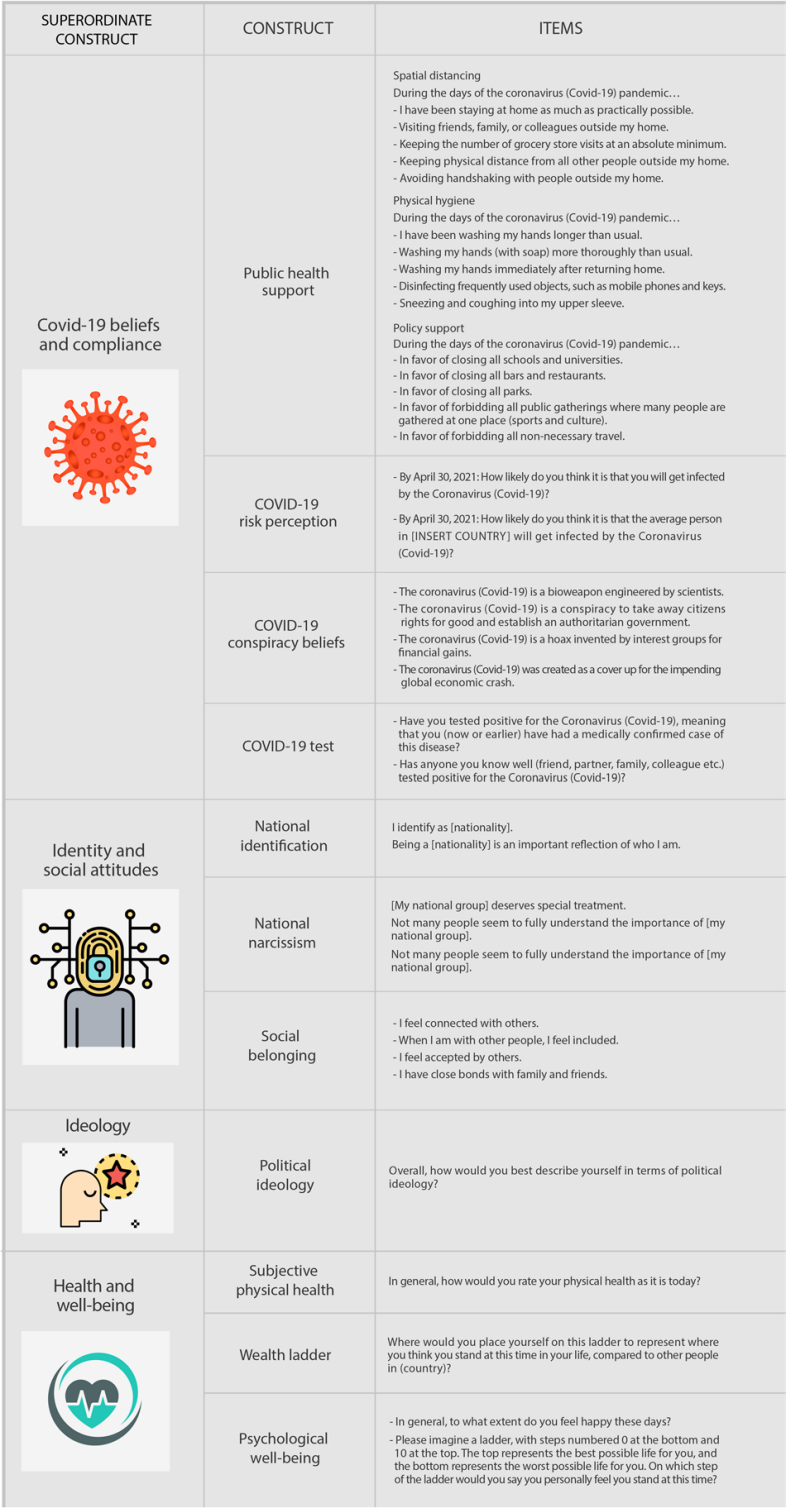
International Collaboration on the Social and Moral Psychology of COVID-19: Investigated constructs, items and variables.

**Fig. 5:**
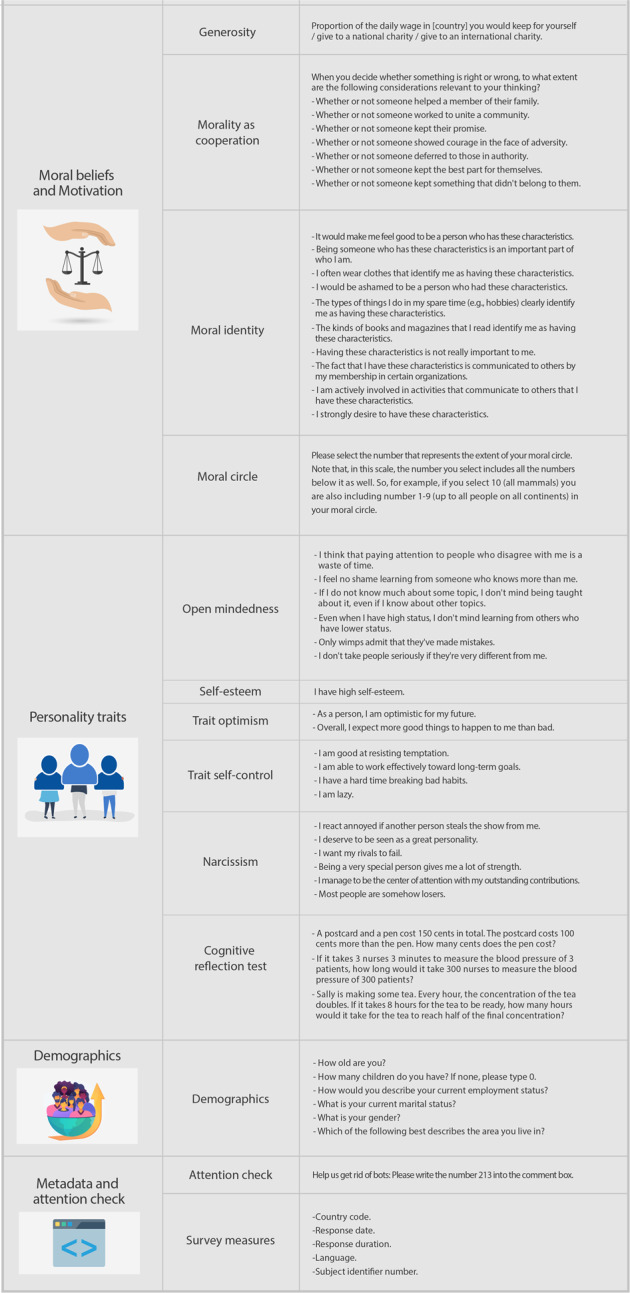
International Collaboration on the Social and Moral Psychology of COVID-19: Investigated constructs, items and variables.

The measures we used are illustrated in Figs. 4, 5 along with the specific items listed for each measure. In most cases, participants’ responses were collected on a scale from 0 = ‘strongly disagree’ to 10 = ‘strongly agree’, with 5 = ‘neither disagree nor agree’. In some cases, when more appropriate, we used other response scales (e.g., the generosity measure, where a 0–100% response scale was applied to hypothetical donations). In total, we collected 98 unique variables and meta-data. To ensure participants’ anonymity, no data that would allow their identification were collected.

#### COVID-19 Beliefs and compliance

Four constructs: COVID-19 public health support, COVID-19 risk perception, COVID-19 conspiracy theory beliefs, and COVID-19 testing behaviour. The public health support construct, in turn, is composed of three measures: spatial distancing, physical hygiene, and policy support. These are ad-hoc scales that we developed ourselves.

#### Identity and social attitudes

Three constructs: national identification^31^, national narcissism^32^, and social belonging^33^.

#### Ideology

One construct: political ideology. Participants self-reported their political orientation according to a single item on a scale from 0 (“Very left-leaning”) to 10 (“Very right-leaning”). This measure has been shown to account for a significant proportion of the variance in voting intentions in American presidential elections between 1972 and 2004^34^ and 2016^35–37^. In fact, using a single-item scale to measure political ideology has been a common practice in political psychology literature, providing substantive evidence for the validity of the measure both across national and international research^38,39^. However, even if the symbolic ideology can be a useful and parsimonious instrument to study political attitudes, when interpreting results, users should be attentive to the political and cultural applicability, psychometric validity, and generalisability of measures of political ideology^40–42^.

#### Health and well-being

Three constructs: subjective physical health, wealth ladder, and psychological well-being. Each of these scales relied on well-validated instruments^43–45^.

#### Moral beliefs and motivation

Four constructs: generosity^46^, morality as cooperation^47^, moral identity^48^, and moral circle^49^.

#### Personality traits

Six constructs: open-mindedness^50^, self-esteem^51^, trait optimism^52^, trait self-control^53^, narcissism^54^, and cognitive reflection^55^.

#### Demographics

Six questions: age, number of children, employment status, marital status, gender, and urbanicity.

#### Metadata and attention check

An attention check was used to mitigate negative impact on data quality from potential non-human responses and the likelihood of biasing data and subsequent analysis of low base-rate outcomes—such as endorsement of COVID-19 conspiracies. We collected typical questionnaire metadata (e.g., start, record, and end dates, duration, and language). In addition, we created an internal participant ID, added ISO2 and ISO3 country codes, and sample representativeness.

### Translation

The survey instrument was drafted in English and translated into other languages using the standard forward-backward method (i.e., members of national teams were advised to split members into forward-translating the survey into the local language and back-translating it into English, and then have the two groups discuss and resolve discrepancies). In total, the survey instrument was translated into 32 languages, including adaptations of region-specific dialects or vernaculars. Specifically, from English into Arabic, Bengali, Bulgarian, Croatian, Danish, Dutch, Finnish, French, German, Greek, Hebrew, Hungary, Italian, Japanese, Korean, Kurdish, Latvian, Macedonian, Mandarin simplified, Mandarin traditional, Nepali, Norwegian, Polish, Portuguese, Romanian, Russian, Serbian, Slovak, Spanish, Swedish, Turkish, and Ukrainian (see osf.io/tfsza at sub-folder *Translations*).

### Data cleaning

We received individual data files from each national team. To merge these raw data, minor modifications were introduced, which we delineate in this section. First, we renamed columns to match across data sets, reordered variables alphabetically, and standardised variable labels. Furthermore, all missing values and values denoting the absence of a response were converted to *NAs* (not available). When ambiguous date formats were found (e.g., on start date, end date, and record date), we manually specified the correct format and standardised them. At the second stage, we introduced multiple modifications to clean the data for research. Some modifications were introduced to every national data set, while others were introduced to specific national data sets (both of which are thoroughly reported in the Data Records section). To each national data set, we recoded the attention check (attcheck) into pass (1) or fail (0); standardised generosity items (generosity1–3), recoded CRT items into intuitive (2), correct (1), and incorrect (0); converted the number of children (children) into a variable with a fixed range from zero to ten or more; recoded all participants declaring being older than 100 years old as 100; and we excluded all duplicates (i.e., in case multiple participants were recorded with identical inputs within a national database, only the first input was retained).

## Data Records

All materials associated with the ICSMP COVID-19 project can be found on the project’s repository (comprising five folders) hosted by the Open Science Framework (OSF, 10.17605/osf.io/tfsza)^56,57^. The folder named *Code* includes an R Markdown document (ICSMP official data.Rmd; osf.io/dwpng) that loads multiple data files (from each national team), cleans them up, merges them into a single data file, generates a data-driven code-book, and saves all outputs. It also includes a reproducible report with all reported numbers, analyses and graphs in this article (Analyses-SciData.html; osf.io/s5c4p; Analyses SciData.Rmd; osf.io/9suyb). The folder named *Data* includes three sub-folders. The *Raw data* sub-folder contains the original and unmodified data files from each national team (country data files.zip; osf.io/dqmut). The sub-folder named *Cleaned data* contains the merged and cleaned dataset, which is provided in a non-proprietary (ICSMP_cleaned_data.csv; osf.io/ypkrc) and a labelled (ICSMP_cleaned_data.sav; at osf.io/8tyj9) file formats. In addition, we included in a sub-folder a dataset that removes observations failing the attention check or filled out less than 50% of the items, both in a non-proprietary (ICSMP_cleaned_data_nobots.csv; osf.io/98fex) and a labelled (ICSMP_cleaned_data_nobots.sav; at osf.io/3yjga) file formats. The *Metadata* sub-folder provides a thorough itemised description of the data cleaning process in both text (Data Cleaning.docx; osf.io/7udpt) and human-readable change-log (human-readable change log ICSMP.xlsx; osf.io/fydx2).

We also provide a data-driven codebook detailing how each measure was collected—e.g., listing variable names, variable labels, and label values (dt.codebook.xlsx; osf.io/ecva2). The *IRB* folder contains both the Internal Review Board Ethics application (ICSMP Kent Ethics application full.pdf; osf.io/xt9gr) and Ethics approval (ICSMP Kent Ethics approval.pdf; osf.io/ce638). The folder *Sample Type & Representativeness* includes the documentation for an internal survey conducted with national team leaders about the employed survey methodology for the data provided (Sample Type & Representativeness.zip; osf.io/fj5xn). The folder *Survey Instrument* contains the initial English version of our survey instrument along with its Qualtrics.qsf for reproducibility (Survey Instrument.zip; osf.io/nf48q). In the sub-folder *Translations*, we archived all 32 translated survey instruments along with a report on the languages of conducted surveys per country (i.e., several countries had their surveys in multiple languages per country; Country and language.xlsx; osf.io/wj7d2).

### Potential for future research

The data contains four measures of COVID-19 beliefs and compliance, 17 social and moral psychological constructs, and six sociodemographic characteristics, amounting to 27 socially-relevant variables. To quantify the potential of this dataset—and assuming a typical research paper uses between three to five key main constructs plus sociodemographics and controls—we calculated the number of combinations of 17 constructs, taken three, four, and five at a time, yielding a grand total of 9248 possible unique designs. As a demonstration of the broad scope of the ICSMP data, published studies cover a broad range of psychological disciplines, including social psychology^13,14^, cognitive psychology^15,17^, political psychology^16^, moral psychology^16,18^, economic psychology^19^ and health sciences^20^, among others. They explore different populations in reference to the COVID-19 pandemic in terms of age (e.g., older adults see^21^, marital status^19^ or nationality (e.g., for a study on the Spanish population, see;^22^ for Swedish and Chinese population see^23^), and other socio-demographic characteristics. These all attest to the great potential of the ICSMP data to inspire further research. In sum, the present dataset affords numerous opportunities for cross-cultural research on a plethora of hypotheses. We encourage researchers who consider reusing ICSMP data to examine the list of pre-registrations before beginning a new project so as to avoid duplication (see icsmp-covid19.netlify.app/preregistration).

### Data visualisation interface

In addition to the raw data, a dedicated Web application was developed to provide a general overview of the dataset (icsmp.shinyapps.io/icsmp_covid19). The application is based on an R shiny server (rstudio.com/products/shiny), together with the *leaflet*^58^ and *ggplot2*^59^ graphical libraries to generate dynamic plots. All the generated figures can be exported as .png files, and all tables can be exported as .csv files. The Web application allows easy and dynamic generation of illustrations like the figures with maps for each construct with zoomable world maps and static figures and plots for sample and country characteristics. In addition, all tables are embedded with dynamic features for sorting and filtering. To make it more accessible for the readers, both tables and figures are downloadable. The Shiny app has two tabs giving general information about the project and the international consortium. The first tab contains sample descriptions such as sample size, missing data, and attention checks for each country with a Gantt chart showing the dates of data collection. The second tab displays world maps of spatial distancing, policy support, national identity, conspiracy beliefs, national narcissism and morality as cooperation as well as all tables reported in dynamic formats.

## Technical Validation

To support the technical quality of the dataset, we conducted an analysis to showcase its reliability (and its diverse applicability to research questions in social sciences and beyond). For completeness, in the analyses that follow, we examined all samples-including those with very few observations, such as Puerto Rico (N = 2), Brazil_3 (N = 6), and Panama (N = 12).

We evaluated the adopted survey methodology utilised by national teams by conducting an internal survey to ensure the accuracy of reported sample types. The inspection showed that 28 samples were quota-based nationally representative samples (36%), 6 used *post hoc* weights to achieve an approximate level of national representation (8%) which nonetheless should be seen as convenience samples, and 43 were convenience samples (56%), many of which were from low and middle-income countries^60^. We codified the results of this survey into the cleaned data as the variable ‘sample_coding’ and present a summary in Table 10. National representativeness for the 28 quota-based samples relate to an approximation of the demographic characteristics of age and gender only for each country.

**Table 10:** Overview of the samples.

Sample Coding	Samples (Countries)	N Samples	N Respondents	% Countries	% Respondents
Continued on next page Quota-based nationally representative	AU, BR_1, CA_e, CA_f, CH, CN, DE, FR, HR, HU, IL, IT_1, JP, KR, LV, NG, NO, NZ, PH, PL, RO_1, RU, SG, SK, TR, TW, GB, US	28	26173	0.36	0.51
Post-hoc weights	AT, DK, ES, NL, SE, UA	6	6703	0.08	0.13
Convenience	AE, BD, BE, BG, BR_2, BR_3, CO_1, FI, GH, GR, IE, IN_1, IN_2, IT_2, IQ, CO_2, AR, CL, MX_2, PE, VE, CR, PY, BR_3, EC, GT, UY, BO, SV, PA, HN, CU, NI, DO, PR, MA, MK, MX_1, MX_2, NP, PK, RO_2, RS, SN, ZA	43	18528	0.56	0.36
Total		77	51404	1.00	1.00

**Fig. 6:**
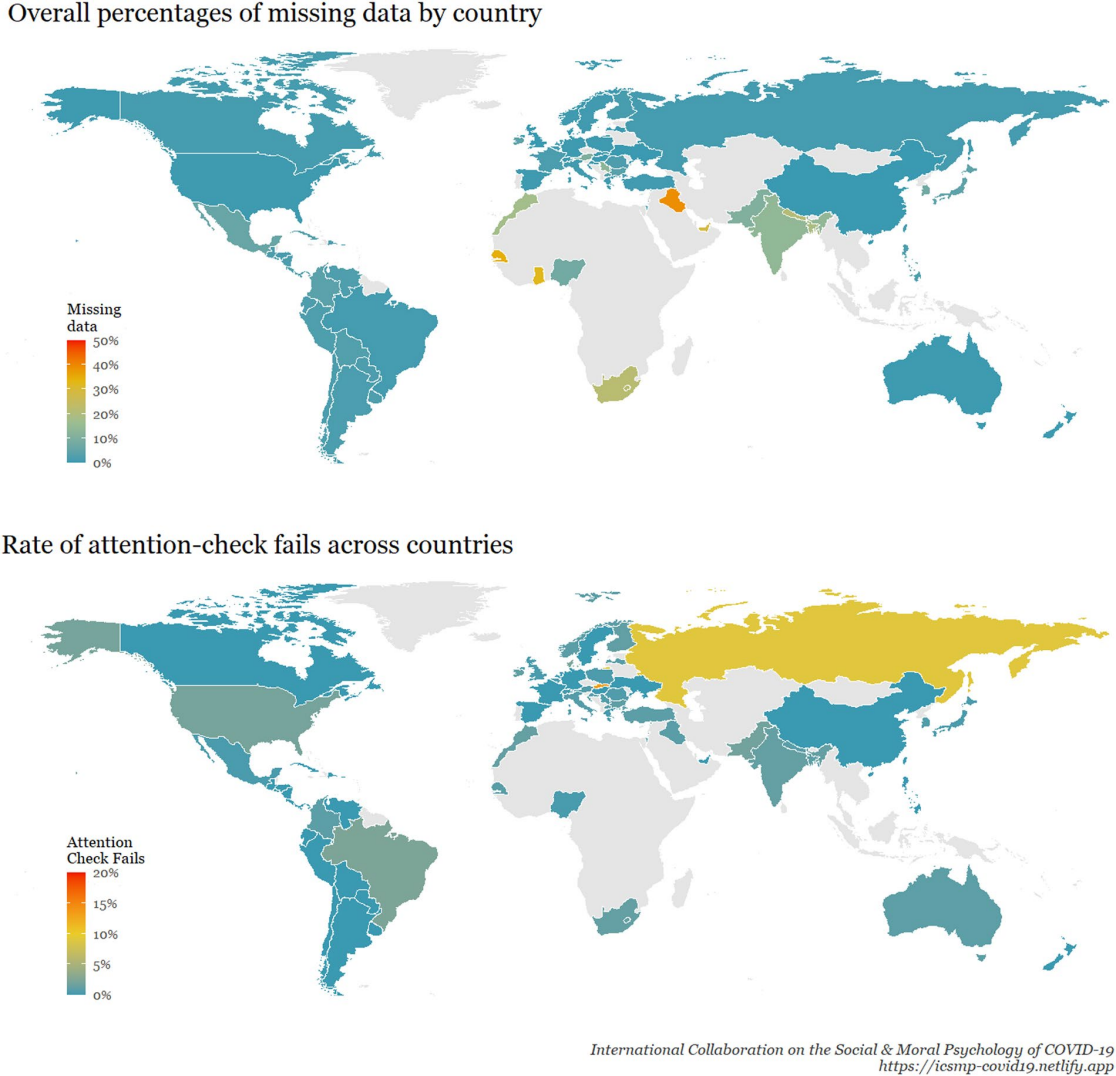
Data quality indicators for each surveyed country. ***Note:*** The percentage of missing data considered all the questions in the survey (i.e., all sociodemographics and psychological scales”). We calculated, for each country, the mean of the participants’ proportion of missing data across all survey questions, including sociodemographics (this information is also provided in our reproducible report of Fig. 6, where the R code is provided).

Regarding individual-level data quality, Fig. 6 shows a world map of the 69 countries from which data were collected, coloured according to overall percentages of missing data (overall mean = 6.0%). Overall, 95.6% of participants had less than 50% missing data, 92.8% participants had less than 10% missing data, and 24.7% of participants had 0% missing data. Another indicator of data quality is the rate of attention check fails per country. On the last screen of the survey, participants were given the following instructions: “Help us get rid of bots: Please write the number 213 into the comment box.” Participants who wrote “213” were coded as passing the attention check, participants who wrote anything else were coded as failing the attention check, and those who did not reach this screen of the survey were coded as missing data. Figure 6 also shows (bottom plot) a world map coloured according to the rate of attention-check fails across countries. Overall, 90.1% of participants passed the attention check (1.0% failed), and 8.0% did not reach the final screen with the attention check.

The full dataset presents *N* = 51,404 cases across 69 countries (from 77 samples, 28 of which are quota-based nationally representative), with an average sample size of 745 (SD = 549) and a proportion of valid answers of 95%. The mean age of respondents was 42.93 (SD = 16.04) years, and 50.9% were women (44% males, 0.3% others, and 4.8% unreported). The employment status breakdown shows 44.8% employed full-time, 10.6% part-time, 8.1% unemployed, 10% students, 10.1% retired, 11% other, and 5.3% unreported. The overall marital status shows 33% of respondents were single, 18.7% in a relationship, 42.7% married, and 5.5% unreported. The majority of our participants reported having no children (41.6%), with 16.7% having one child, 20.1%, 9.2%, and 3.9% with two, three and four children, respectively, and 1.7% had five or more children (6.9% unreported). We break down these aggregated results per country. Tables 1, 2 show the number of cases and valid answers, Table 3,4 summarises the distribution of sex, Tables 5, 6 display employment status, and Tables 7–9 illustrate both marital statuses and the number of children.

**Fig. 7:**
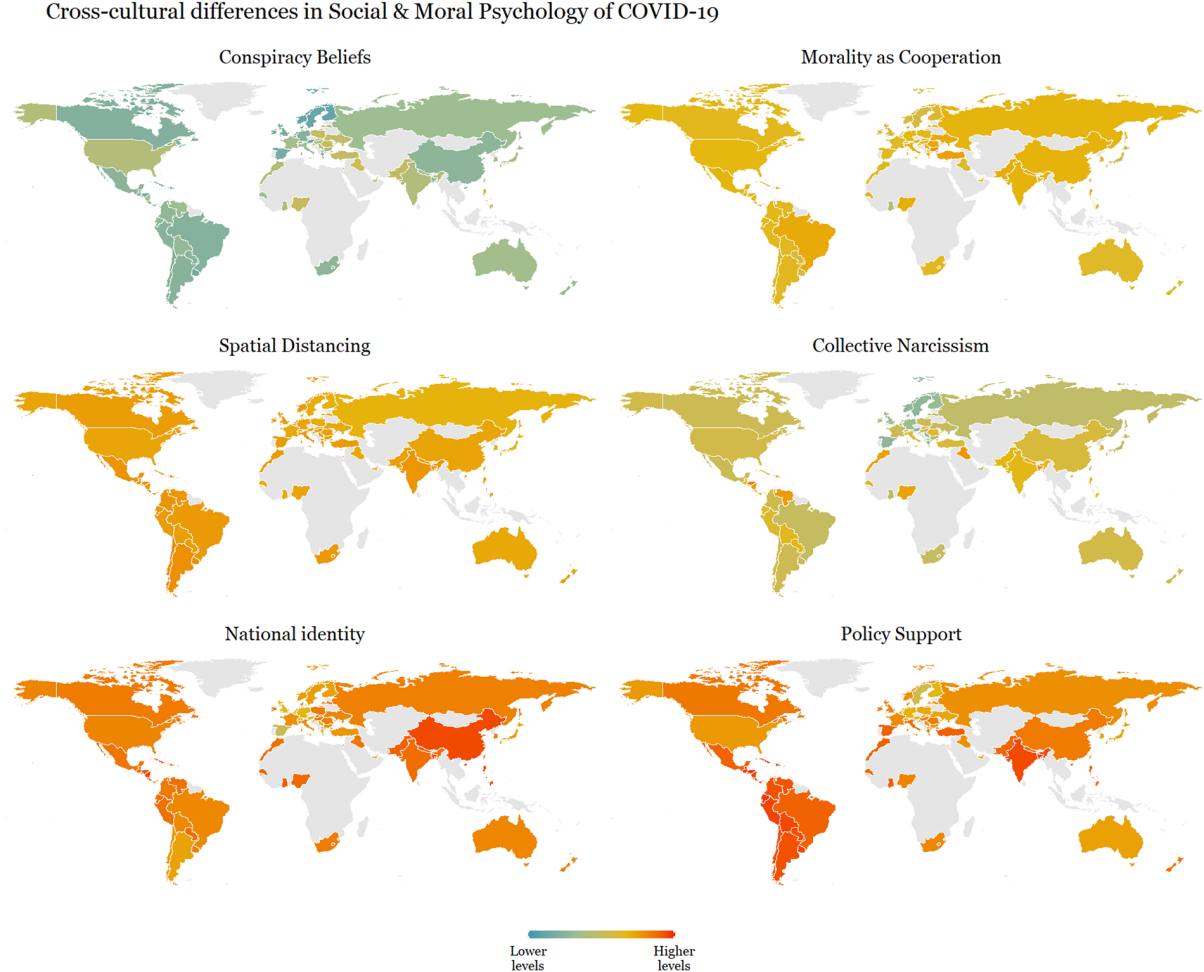
Cross-cultural differences in Social & Moral Psychology of COVID-19 across 69 countries. ***Note:*** Each world heat map in the figure shows the means score, at the country level, for constructs in the survey. Conspiracy Beliefs - participant’s beliefs in conspiracy theories regarding COVID-19; Morality as Cooperation - participant’s moral concern based on the morality-as-cooperation theory; Spatial Distancing - participant’s support for spatial distancing as a strategy against COVID-19; Collective Narcissism - participant’s narcissism, i.e., an inflated view regarding their ingroup (in this research we focused on nationality); National Identity - participant’s identity attached to belonging to a nation; Policy Support - participant’s support to public policies (e.g., closing parks or schools) as a strategy against COVID-19.

**Fig. 8:**
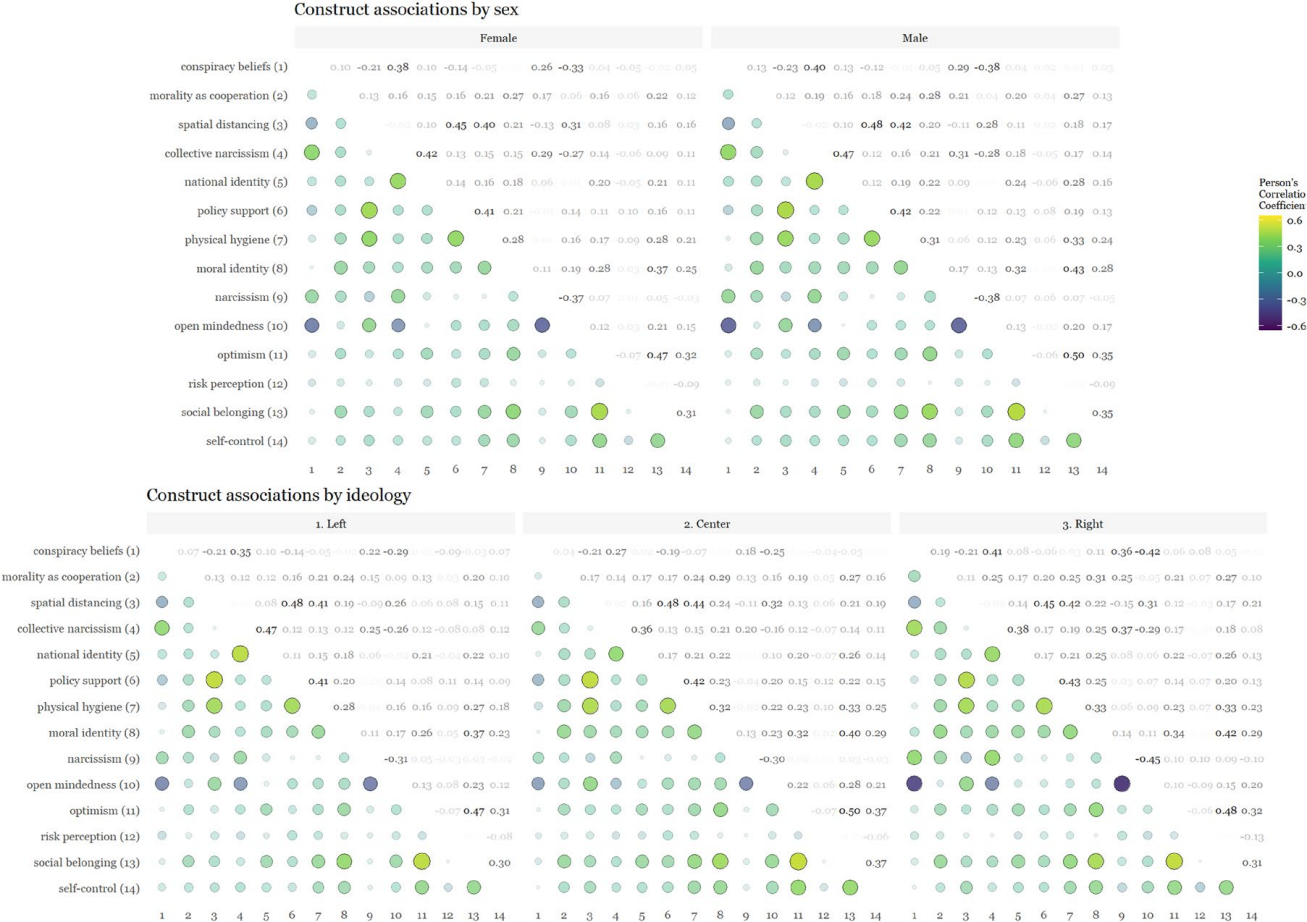
Cross-cultural differences in associations of Social & Moral Psychology of COVID-19 across sex and ideology in 69 countries.

**Fig. 9:**
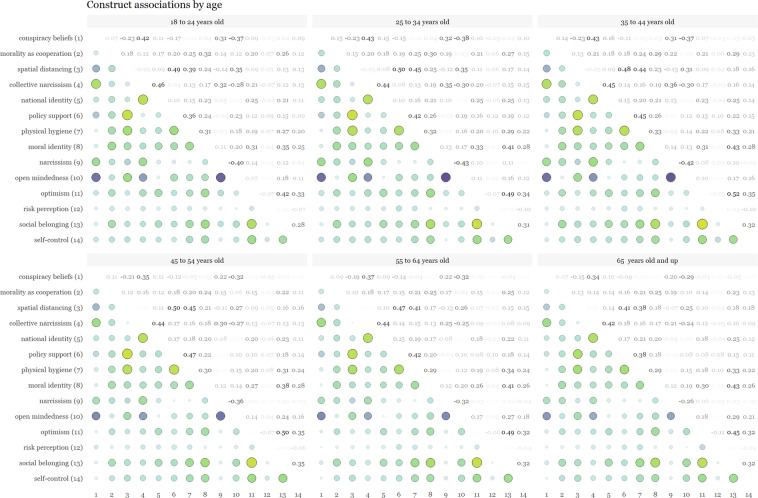
Cross-cultural differences in associations of Social & Moral Psychology of COVID-19 across age in 69 countries.

We also examined cross-cultural differences in conspiracy beliefs, morality as cooperation, spatial distancing, national narcissism, national identification, and policy support for preventative measures across 69 countries in Fig. 7. Additionally, we showcase patterns of associations between these moral and psychological constructs across gender, ideology and age in Figs. 8, 9. For the association pattern analysis, we excluded samples with less than 490 respondents as recommended for stable correlations^61^, as well as for the subsequent consistency measure analysis.

**Fig. 10:**
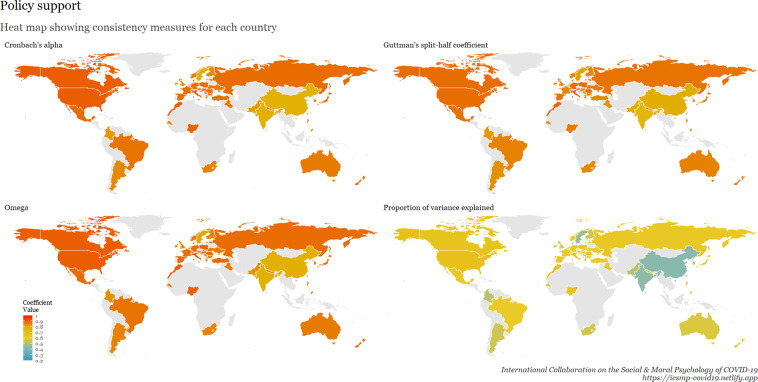
Support for policies in 69 countries.

**Fig. 11:**
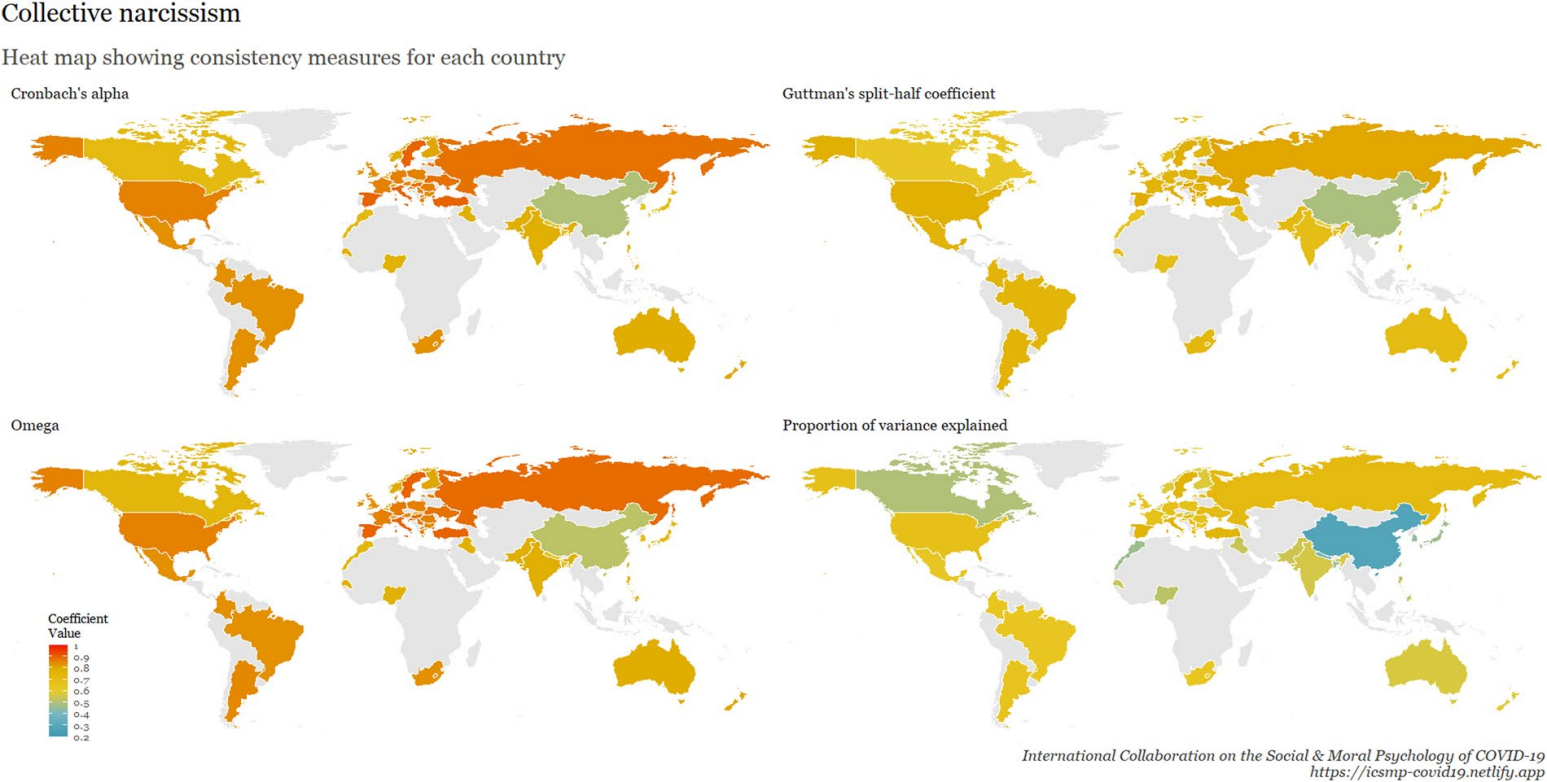
Collective narcissism in 69 countries.

**Fig. 12:**
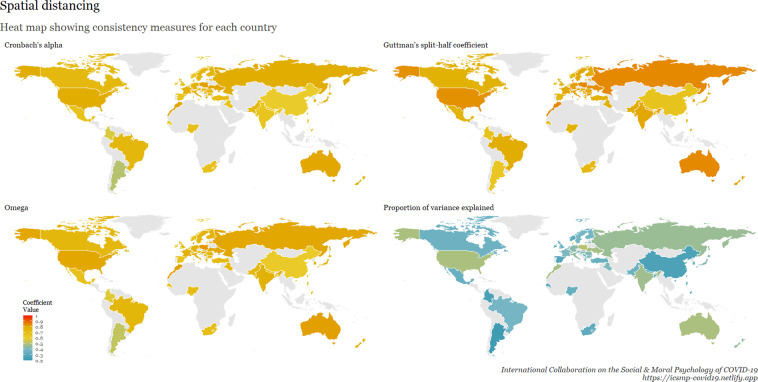
Spatial distancing in 69 countries.

**Fig. 13:**
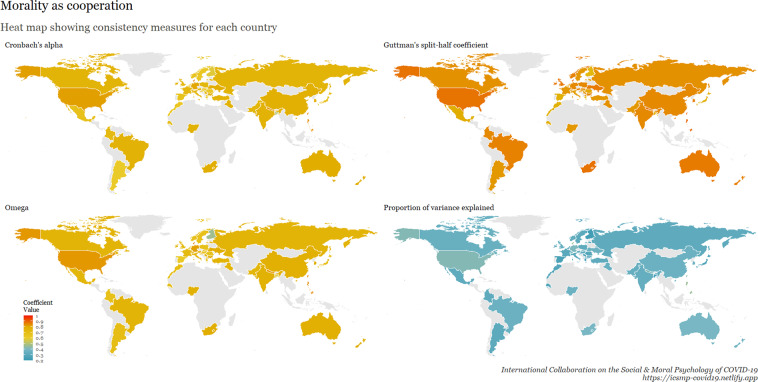
Morality as cooperation in 69 countries.

**Fig. 14:**
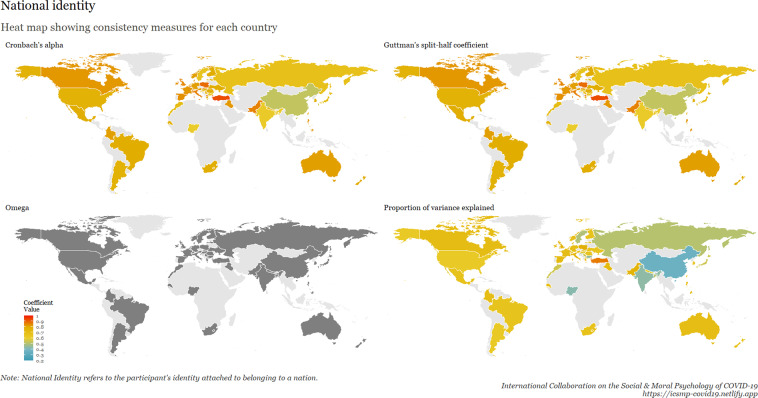
National identity in 69 countries.

**Fig. 15:**
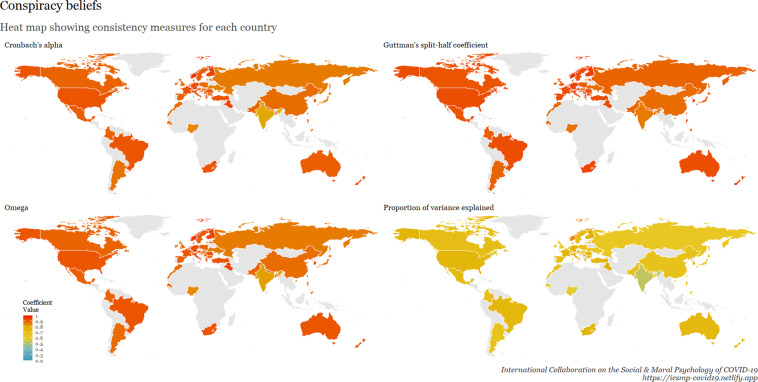
Cross-cultural differences in Internal Consistency Coefficients (Cronbach’s alpha, McDonald’s Omega, Guttman Split-Half), and variance explained of Social & Moral Psychology Constructs in 69 countries. ***Note:*** internal consistency typically refers to correlations between different items on the same test to evaluate the extent to which latent indicators comprising the scale measure the same construct.

To examine internal consistency for the main scales, we calculated Cronbach’s Alpha, Omega, Guttman split-half reliability, and proportion of variance explained by a unidimensional factor. This table is available at osf.io/ed7yg and shows indices of internal consistency by country for measures of conspiracy beliefs, morality as cooperation, spatial distancing, national narcissism, national identification, and policy support for preventative measures, respectively. We found that the spatial distancing construct, on average, has the lowest Cronbach’s alpha, followed by morality as cooperation. On average, conspiracy beliefs have the highest Cronbach’s alpha, followed by policy support. These patterns hold for the Omega measures, but when considering Guttman’s split-half reliability, collective narcissism and national identity yield the lowest values. Figures 9–15 show these patterns visually.

## Usage Notes

The datasets are shared, cleaned, and ready for analysis. We recommend that interested researchers use the cleaned version of the data (available at 10.17605/osf.io/tfsza)^56^. The use of the labelled data is also suggested for convenience as it has all variable levels encoded, thus eliminating the need to consult the codebook when using the.csv format.

The Data were imported and cleaned using the R software for statistical analysis^62^ and packages *readr*^63^, *haven*^64^, *readxl*^65^, *dplyr*^66^, *psych*^67^, *htmltools*^68^, mime^69^, *xfun*^70^, *labelled*^71^, *sjlabelled*^72^, *codebook*^73^, *lubridate*^74^.

As previously noted^5^, those wishing to approximate national representativeness can apply the appropriate survey weights to demographic and countries of interest when random sampling is used (e.g., sex: https://ourworldindata.org/gender-ratio; age: http://data.un.org/Data.aspx?d=POP&f=tableCode%3A22; education: https://ourworldindata.org/global-education; marital status: https://ourworldindata.org/marriages-and-divorces).

To minimize misclassification of text-based responses to the cognitive reflection test (CRT) and the attention check, we used multiple steps of data cleaning using REGEX (regular expressions) as fully detailed in (ICSMP official data.Rmd; osf.io/dwpng) located in the folder named *Code*. First, we coded the predefined numerical and text values as correct (in the case of CRT, also the values predefined as intuitive). Then, iteratively, we screened the remaining responses and, using REGEX, updated answers. Remaining responses were recoded as incorrect.

## Data Availability

All raw and cleaned data—as well as the R-code—used for standardising national-teams data, merging, and cleaning them are available at 10.17605/osf.io/tfsza^56^.
